# eDNA sampled from stream networks correlates with camera trap detection rates of terrestrial mammals

**DOI:** 10.1038/s41598-021-90598-5

**Published:** 2021-06-15

**Authors:** Arnaud Lyet, Loïc Pellissier, Alice Valentini, Tony Dejean, Abigail Hehmeyer, Robin Naidoo

**Affiliations:** 1 World Wildlife Fund, Wildlife Conservation Team, Washington, DC USA; 2 World Wild Fund for Nature, Tigers Alive Initiative, Singapore, Singapore; 3 Landscape Ecology, Institute of Terrestrial Ecosystems, Department of Environmental Systems Science, ETH Zürich, Zürich, Switzerland; 4 Swiss Federal Institute for Forest, Snow and Landscape Research WSL, Birmensdorf, Switzerland; 5 SPYGEN, Le Bourget du Lac, France; 6 University of British Columbia, Vancouver, BC Canada

**Keywords:** Biodiversity, Conservation biology, Next-generation sequencing, High-throughput screening

## Abstract

Biodiversity monitoring delivers vital information to those making conservation decisions. Comprehensively measuring terrestrial biodiversity usually requires costly methods that can rarely be deployed at large spatial scales over multiple time periods, limiting conservation efficiency. Here we investigated the capacity of environmental DNA (eDNA) from stream water samples to survey terrestrial mammal diversity at multiple spatial scales within a large catchment. We compared biodiversity information recovered using an eDNA metabarcoding approach with data from a dense camera trap survey, as well as the sampling costs of both methods. Via the sampling of large volumes of water from the two largest streams that drained the study area, eDNA metabarcoding provided information on the presence and detection probabilities of 35 mammal taxa, 25% more than camera traps and for half the cost. While eDNA metabarcoding had limited capacity to detect felid species and provide individual-level demographic information, it is a cost-efficient method for large-scale monitoring of terrestrial mammals that can offer sufficient information to solve many conservation problems.

## Introduction

Data quality plays a critical role in detecting and understanding biodiversity change (e.g. ref.^1,2^), but generating information over entire landscapes with robust and repeatable sampling is generally outside the scope of most management and conservation programs^3^. The taxonomic groups most often in need of conservation efforts, such as vertebrate predators and their prey^4–7^, are also the most elusive and difficult to monitor. As a result, conservation decisions are often made using outdated, static and/or fragmentary data that do not necessarily reflect how species use the landscape, and are therefore more likely to fail^8,9^. For decades, the need for higher quality biodiversity information balanced with budget, time and capacity constraints has motivated the search for more cost-efficient methods and optimal sampling designs for biodiversity across wide landscapes^10^. Efficient sampling designs would enable balancing the benefits of collecting presence or abundance data at a large number of sites with the costs of field sampling^11^. While it remains a challenge, continuing technological advances suggest that trade‐offs between sampling precision, sample size and the extent of the sampling area^12^ might soon be transformed by novel biomonitoring methods.

The gap in both spatial and temporal biodiversity data for conservation could be filled by environmental DNA metabarcoding approaches (eDNA hereafter)^13^, which enables the capture, amplification and sequencing of DNA molecules from aquatic environments that allow the inference of species presence^14^. Networks of streams, through which precipitation percolates, function as conveyor belts of DNA from the entire landscape^15^.While eDNA from stream water has mainly been used to detect aquatic organisms^16^, it also offers the potential for detecting terrestrial organisms^17,18^, and could be used for quantitative assessment of terrestrial biodiversity. A deeper understanding of the detectability of terrestrial organisms via stream eDNA would allow more efficient use of this approach for monitoring biodiversity and conservation^19,20^. Unlike aquatic animals whose DNA is inherently in the water, the mechanisms by which the DNA of a terrestrial animal is transferred to stream water are complex and likely depend on the behaviour of the animal^21^. Where the animal goes and what it does affects the quantity and quality of interaction with water, for example swimming, wallowing, release of saliva while drinking, and deposition of urine or faeces in water^22–25^. Ecological factors such as the abundance and distribution of the species, and its position in the food chain—faeces dropped in water will transfer DNA of the host species and of all animal species consumed^26^—might also affect the detection probability of taxonomic groups within water eDNA samples. When combined with environmental properties of hydrological networks such as the size of the stream^27^, the drainage area or the amount of precipitation, it is clear that a variety of factors could influence terrestrial species detectability via eDNA^17,28^.

**Figure 1:**
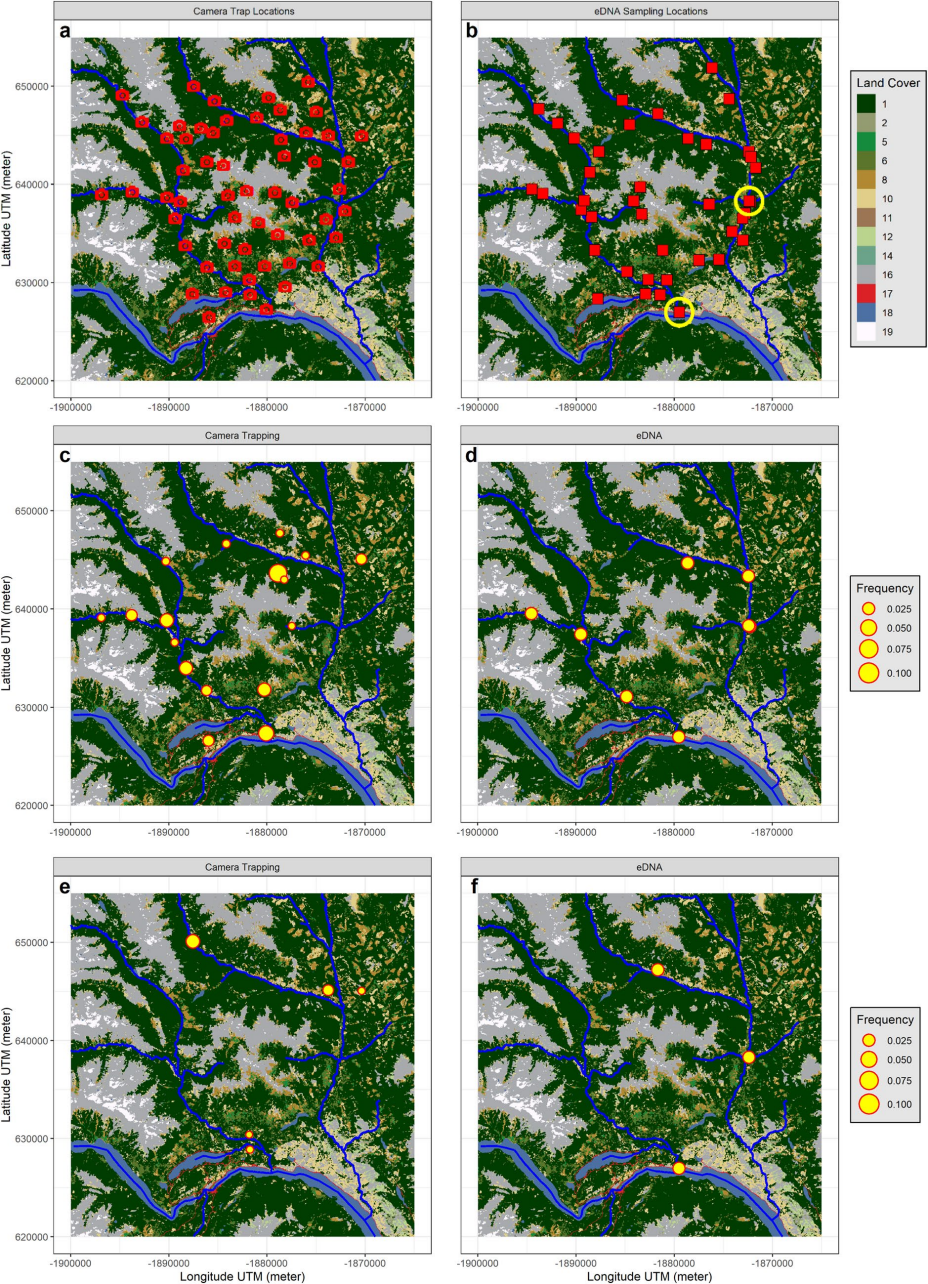
Study area, sampling location and species detection examples. The top of the figure indicates North and the scale is given by the latitude and longitude graduations shown in Universal Transverse Mercator (UTM) in meter (see Supplementary Figure 1 for the general location of the study area in British Columbia). The top row panel (**a**,**b**) shows the sampling sites within the study area's hydrological system, the camera trap (**a**) and the eDNA sampling locations (**b**) in 2018 (red squares) and 2019 (yellow circles). The main streams are shown on the maps (blue lines) as well as the main Land Cover types. Code descriptions: 1, 2, 5, 6 = forest; 8, 11 = grasslands and 10, 12 = shrublands; 14 = wetland, 16 = barren lands; 17 = urban; 18 = water and 19 = snow and ice. The UTM coordinates in meter are shown on both x and y axis. The two bottom rows (**c**–**f**) show the comparison of camera trap 2018 (left) vs. eDNA 2018 (right) detection of two species, *Alces alces* (**c**,**d**), and *Martes pennanti* (**e**,**f**), in the study area. For the camera trap method, the size of the circle indicates the average number of independent detections per day at the station. For eDNA, the circle only indicates that the species was detected in at least one sample at the location. The maps were created using R v4.0.3^55^ (https://cran.r-project.org/).

Here, we evaluate the opportunities that eDNA offers for understanding the spatial and temporal distribution and detection probabilities of terrestrial species, and for documenting multiple facets of biodiversity at the landscape scale. We propose a catchment-level eDNA sampling framework directed towards multispecies monitoring programs, and test it against conventional surveys of the terrestrial mammal assemblage in a mountainous landscape in British Columbia, Canada. We evaluate the effectiveness of two different eDNA survey designs based on the sampling of large volumes of stream water: a *spatial design* allowing fine scale site-level monitoring vs. a *catchment design* sampling from two streams at the base of the catchment (Fig. 1). By contrasting these two sampling approaches, we investigated the ability of eDNA to capture diversity at different spatial scales: at sites, between sites, and across the entire landscape. We compared the eDNA surveys with a simultaneous camera trap survey covering the same area, and with prior knowledge of the regional mammal community. We hypothesized that species ecology would play a strong role in shaping the effectiveness of eDNA surveys for terrestrial mammals: semi-aquatic and small mammals that are abundant and represent a large proportion of the diet of small carnivores^29^ and birds of prey^30^ would be amongst the most frequently detected groups, followed by herbivores and omnivores which produce larger quantities of faeces and are more abundant^29^. In contrast, we expected that carnivores at the top of the food chain would be more difficult to detect as they are present at lower abundance and must drink from or enter a body of water for eDNA deposition to occur^23–25^. Moreover, in addition to species' life history attributes, we expected that physical characteristics and environmental conditions of the stream channel sampled, including catchment area, stream size and precipitation, would all further influence the signal recovered from terrestrial mammals via eDNA.

## Results

The eDNA surveys involved filtrating a total of ~ 1,400 L of water in 2018 (50 samples from 42 sites) and ~ 2,300 L in 2019 (36 samples from 2 sites). We obtained a total of 12,757,213 sequencing reads in 2018 and 7,641,854 reads in 2019, of which 6,310,334 (49.5%) and 4,308,349 (56.4%) were retained, respectively, after bioinformatic filtering (see “Methods” section). The camera trap monitoring^31^ involved 57 motion-triggered camera traps that were active for a total of 5,746 trap-days in 2018 and 5,847 in 2019. They produced a total of 17,939 independent pictures of mammals in 2018 and 17,946 in 2019.

### Relative landscape-level frequency

**Figure 2:**
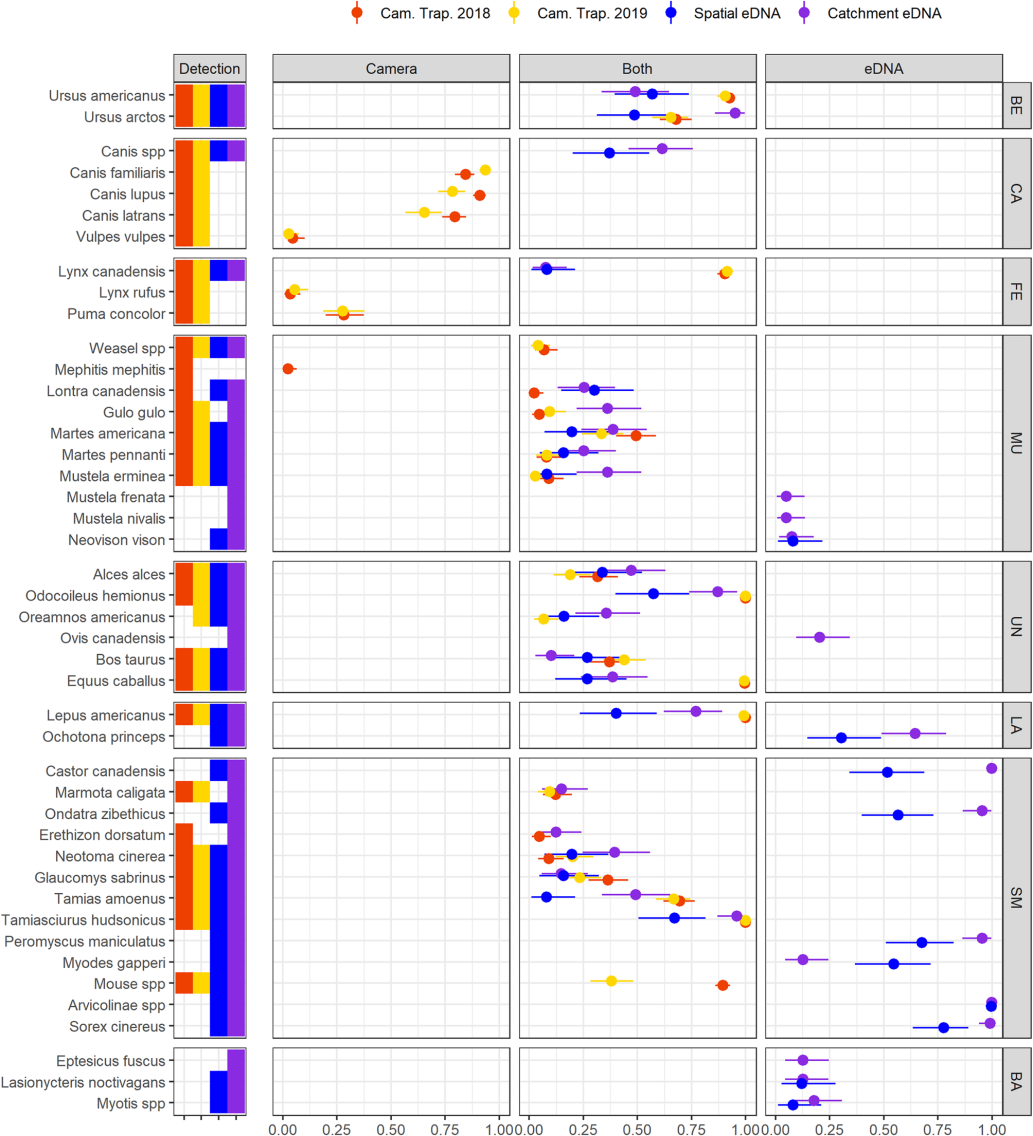
Overall species detection probabilities. To highlight the differences between methods, we divided the figure in three panels labelled “Camera”, “Both”, and “eDNA”. The left panel shows species detected via camera trapping only, the middle panel shows species detected by both camera trapping (red and yellow) and either eDNA sampling strategy (blue and purple), and the right panel shows species detected only by eDNA. The plain circles indicate the average detection probabilities for every species. The horizontal bars show the limit of the 95% Bayesian Credibility Interval. Scientific names of the taxa are on the left of the panel. Taxa are grouped by higher taxonomic level, which code is displayed on the right of the figure: BE for Bears, CA for canids, FE for felids, UN for ungulates, LA for lagomorphs, SM for small mammals, and BA for bats. The figure does not include 3 additional species (*Rangifer tarandus*, *Mus musculus* and *Felis* spp) each detected in at least one eDNA sample, but which detection might result from contamination (see supplementary Tables 1a,b).

eDNA sampling effectively characterized mammal assemblage properties at our study landscape. In terms of total diversity, across all sites, field replicates and years, eDNA recorded 32 species-level detections, two genus-level detections, (*Canis*, *Myotis*) and one subfamily-level detection (*Arvicolinae*). Camera traps identified 27 unique species across all years and pictures (26 in 2018, and 24 in 2019) and captured small rodents and mustelids that could not be identified to the species-level (Fig. 2). eDNA sampling uniquely detected and identified 14 taxa (Fig. 2)—12 species-level, one genus-level (*Myotis*) and one subfamily-level (*Arvicolinae*)—but missed the detection of four species of carnivores: the commonly-detected *Puma concolor* (31 pictures in 2018 and 29 in 2019), as well as *Vulpes vulpes*, *Lynx rufus*, and *Mephitis mephitis* which were amongst the least frequently detected mammals (respectively 3, 3, and 1 pictures in 2018 and 1, 3, and 0 pictures in 2019). *Canis* spp were detected in 9 eDNA samples in 2018 and 23 samples in 2019, but the 12S marker used could not differentiate between *C. lupus lupus*, *C. lupus domesticus* and *C. latrans*.

**Figure 3:**
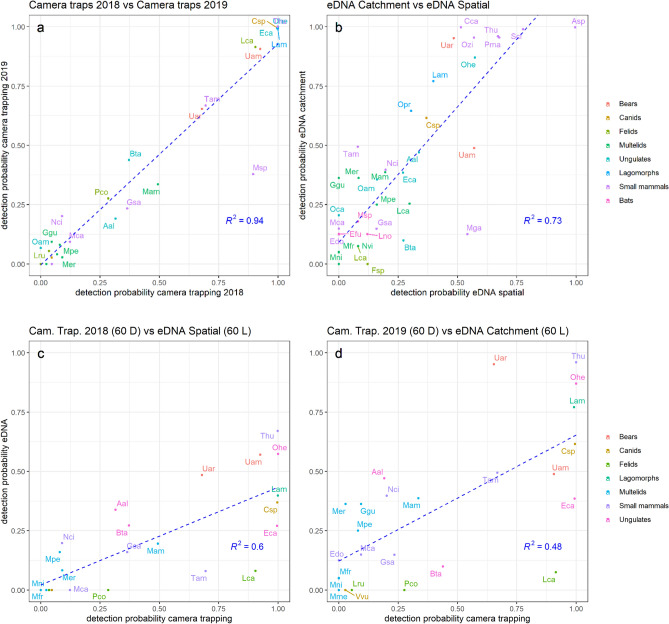
Species detection correlations between years and between methods. The top panels show the comparison of the average detection probabilities between 2018 and 2019 camera trapping surveys (**a**), and between catchment (y-axis) and spatial (x-axis) eDNA sampling strategies (**b**). Panel (**a**) includes only species targeted by the camera trap surveys, while panel (**b**) includes all species. The bottom panels (**c**,**d**) show the comparison of average detection probabilities between camera trapping (x-axis) and eDNA (y-axis), and includes only the species targeted by camera trap surveys: panel (**c**) shows camera trapping 2018 vs spatial eDNA, and panel (**d**) shows camera trapping 2019 vs catchment eDNA. Each individual species is represented by a dot and its associated label corresponds to the first letter of the genus followed by two first letters of the species name (e.g. “Uam” for *Ursus americanus*, or “Csp” for *Canis* spp). Full taxonomic names can be cross-referenced with the y-axis of Fig. 2. The dotted lines show the linear regression between the two variables. R^2^ is the coefficient of correlation of the regression and is shown at the bottom right corner on each panel.

We also found that eDNA sampling performed at the base of the catchments in 2019 (*catchment design*) recovered a higher diversity of species than the sum of local site-level sampling in 2018 (*spatial design*): 32 species and three unresolved taxa for the former versus 25 species and three unresolved taxa for the latter (Fig. 2, Supplementary Table 1a,b). In addition, the detection probability per volume (i.e., detection probability per species per 60 L of water averaged across all samples in the same year; see “Methods” section) was superior or equivalent for all species in the *catchment design* except for *Myodes gapperi.* Despite discrepancies in recovered diversity, we found a strong correlation between eDNA detection probability in the two independent sampling years of 2018 and 2019 (R^2^ = 0.73; Fig. 3) suggesting a robust temporal signal of detection probabilities within eDNA samples.

While eDNA samples for the spatial design and catchment design were collected from different years, the camera trap survey suggested the relative frequency of species present in the study area did not vary between the two years. First, camera detection rates per species were similar between 2018 and 2019 (R^2^ = 0.94), suggesting a stable community of mammals in the study area (Fig. 3, Supplementary Figure 6). Additionally, for species targeted by camera traps, the detection probabilities recovered from both eDNA sampling strategies showed significant positive associations with the average camera trap detection probabilities estimated in 2018 and 2019 (R^2^ = 0.61 in 2018, R^2^ = 0.49 in 2019, Fig. 3, see “Methods” section).

Site-level (or sample-level for *catchment* eDNA) diversity estimates showed more contrasting patterns between eDNA and camera trap survey methods. We found that camera traps recovered a higher diversity per site than eDNA *spatial*, while eDNA *spatial* had a stronger turnover of species among sites, partly explained by differences in local stream environmental conditions or species-specific detection probabilities. The average site-level diversity was 5.6 species for eDNA *spatial*, 15.2 for eDNA *catchment*, 8.8 for camera trapping in 2018 and 8.0 for 2019. The proportion of diversity turnover between sites (or samples for *catchment* eDNA) was 0.66 and 0.68 for camera trapping in 2018 and 2019, 0.81 and 0.57 for eDNA *spatial* and *catchment*, respectively.

### Effect of taxonomic group, local environment, and year on species detection using eDNA

**Figure 4:**
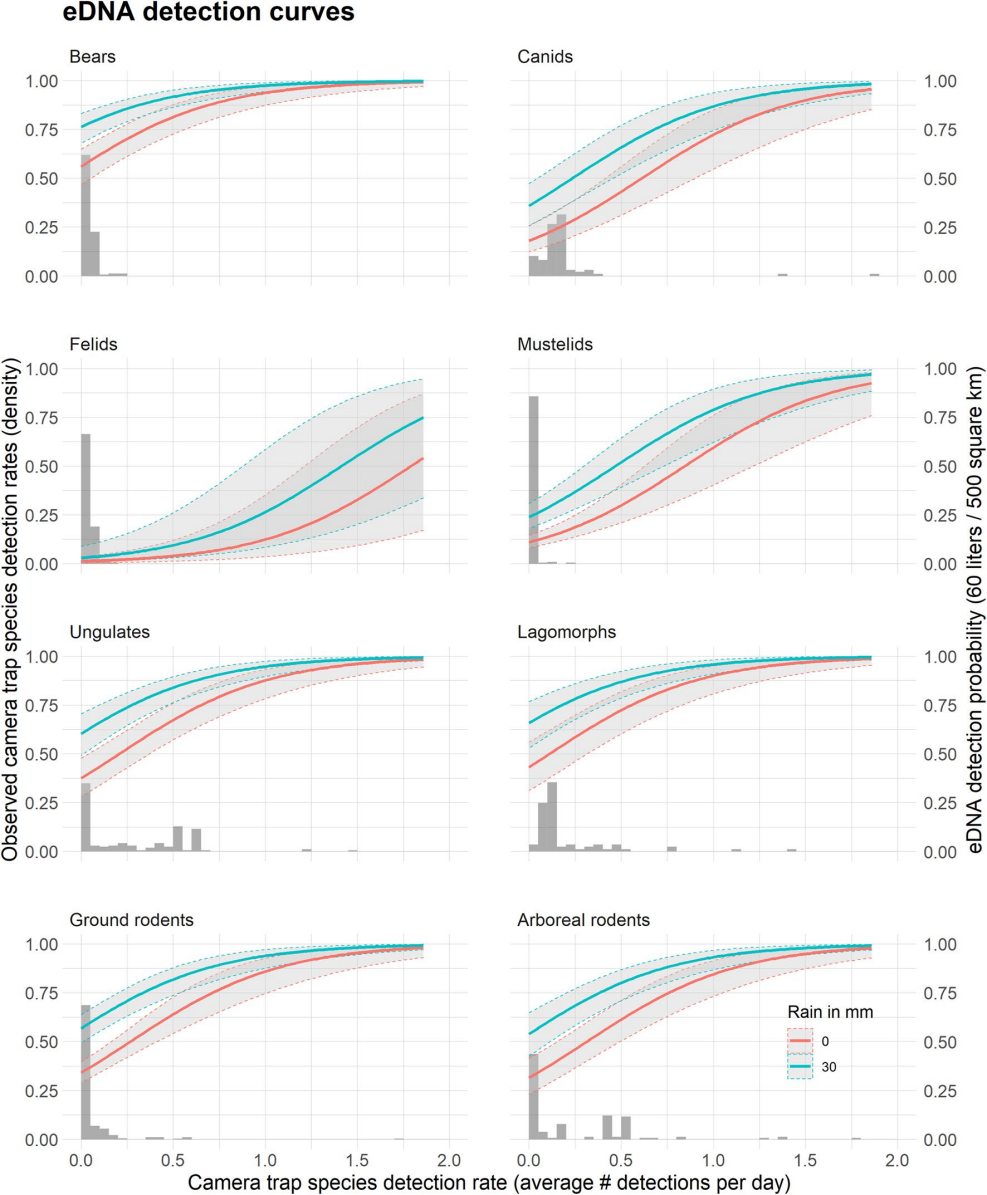
Taxonomic eDNA detection curves. Relationship between the eDNA detection probability at a site (y-axis, right side), the average camera trap detection rate at the catchment (x-axis), and the rain the day prior to sampling (no rain in red, 30 mm in blue). Curves are calculated for 60 L of water filtered from a stream draining a 500 km^2^ catchment. The figure shows the mean predicted value (solid lines) and associated standard errors (± SE, grey areas), calculated using the parameter estimates from the model with the lowest *AICc* (Supplementary Tables 2a,b). The histograms represent the observed density distribution of the average camera trap detection rates associated with eDNA sampling sites (y-axis, left side). For instance, on the “Ground rodents”, the histogram indicates that more than 75% of the eDNA sampling locations were associated with a catchment where on average across all camera trap located in the catchment, less than 0.2 ground rodents were detected every day, or one ground rodent detected every 5 days. Data from 2018 and 2019 are combined.

We found that taxonomic group, species ecology and environmental factors explained differences among detection patterns observed between samples, sites, species and methods. In general, species that were frequently and widely detected on the camera grid were also frequently and widely detected in our eDNA samples, while those rarely detected were also rare in our eDNA samples (Fig. 1, Supplementary Figure 2). We found that species detection probability from eDNA was associated with taxonomy, even when the effect of local environmental conditions and spatial variation patterns in species’ camera trap detection rates were accounted for (see “Methods” section and Supplementary Tables 2a,b). Small mammals had the highest average detection probabilities (0.50 and 0.59 in 2018 and 2019, respectively), followed by lagomorphs (0.34 and 0.67) and ungulates (0.31 and 0.38), with the lowest for carnivores (0.23 and 0.32) and bats (0.10 and 0.14). In addition, we found significant differences in detections between ecological groups related to diet preference, with a higher detection of species with mixed (omnivores) and plant-based (herbivores) diets as compared to species with exclusively animal-based (carnivores) diets (see model selection and parameter estimates in Supplementary Tables 3a,b). The model further indicates that eDNA site detection probability correlates with the catchment area, the volume of water filtered, and precipitation intensity (see taxonomic detection curves Fig. 4), all of which are likely to affect the quality of the information recovered from eDNA. Despite the eDNA catchment and spatial sampling design being implemented in two different years, we found no evidence of a residual year effect that could have been caused by environmental factors not included in our models. In the three model selection procedures performed, the best models that included the year effect were not significantly better than the best model without a year effect. In addition, the beta estimates for this effect were close to zero in all cases, with confidence intervals that included zero (Supplementary Tables 2, 3 and 5).

### Survey cost estimation and comparative cost-efficiency

**Figure 5:**
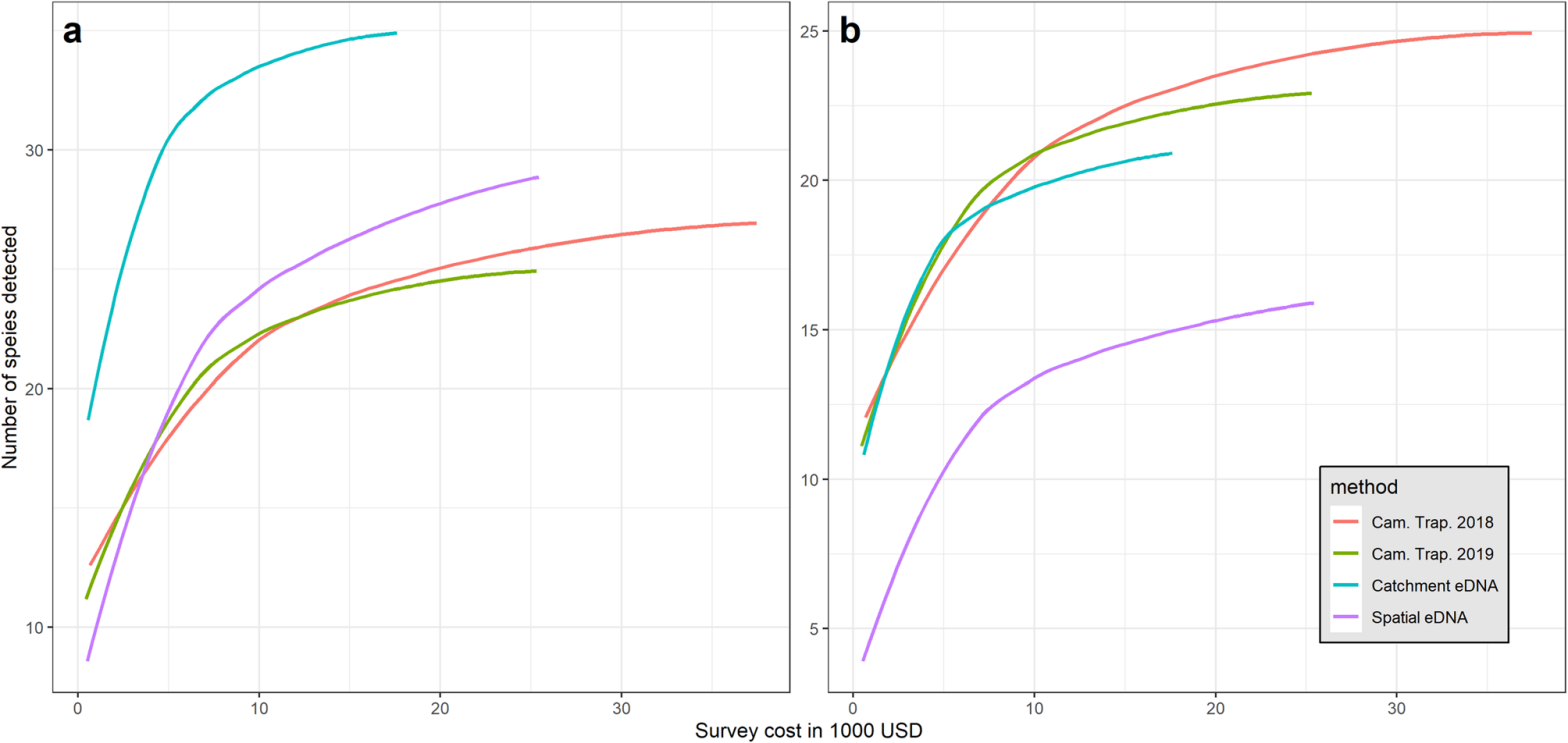
Comparison of cost-efficiency between survey methods to recover the total mammal diversity at the study scale. The figures show the curves of cumulative total mammals’ diversity (y-axis) per thousand USD (x-axis) invested in the survey. Colours indicate the different survey methods and years as shown in the legend. The panel (**a**) compares methods’ cost effectiveness when all the taxa detected by either method are included in the analysis, while panel (**b**) only includes species targeted by camera trapping methods (see “Methods” section). Detailed cost analyses per survey are presented in the method section (see also Table 1 and Supplementary Table 4).

From an estimation of the total cost of each survey type (Table 1, Supplementary Table 4), we assessed the relative cost-efficiency of cameras and eDNA sampling to recover diversity indicators. For each method and year, we investigated the relationship between the number of species detected and the cost of the survey (Fig. 5). We found that the eDNA *catchment design* yields more species detections per dollar invested, while the eDNA *spatial design* and camera trap surveys had similar cost-effectiveness. In particular, the detection of 20 taxa required ten cameras in 2018, fourteen cameras in 2019, ten *spatial* samples of water in 2018, and only two *catchment* water samples in 2019. The respective costs were evaluated at USD $6,710, $6,370, $5,190, and $1,136. Overall, the results indicate that the eDNA *catchment design* is more cost-efficient than the *spatial design* for the assessment of total diversity of species. In addition, our results show that the cost-efficiency of eDNA sampling could be further improved by collecting larger volumes of water than the standard 60 L used to monitor fish^27,32^, and by sampling immediately after heavy rainfall (Supplementary Tables 5a,b, Supplementary Figure 3). Our best model predicts that the filtration of 90 L of water from a large stream (600 km^2^ catchment) after a 30 mm rainfall would yield the detection of 23 species, while 60 L would only yield 19 species.

**Table 1:** Costs of terrestrial mammal inventory for each survey method and year (US $).

Method	# of eDNA samples or camera sites	Re-usable equipment	Single-use equipment	Labour and lab work	Logistic	Total cost	Cost per sample or camera site
CT 2018	57	$11,970	$503	$24,800	$858	$38,273	$671
CT 2019	57	$840	$482	$23,600	$821	$25,922	$455
eDNA 2018	50	$1,053	$4,746	$19,203	$948	$25,950	$519
eDNA 2019	36	$3,498	$2,721	$13,858	$441	$20,465	$568

The table shows a summary of the costs for each survey method and year. Total cost calculation includes four budget components: *re-usable equipment*, *single use equipment*, *labour and lab work*, and *logistic*. *Re-usable equipment* includes camera traps, water pumps and rechargeable batteries, *single use equipment* includes AA batteries and eDNA sampling kits, *labour and lab work* combines staff time for field work as well as time spent for data analysis, *logistic* includes expenses related to local transportation from headquarters to field study sites. Cost per eDNA sample or camera trap site is obtained by dividing the total cost by the number of samples or sites. See Supplementary Table 4 for detailed description of each cost component.

## Discussion and conclusions

Due to the fractal nature of stream networks^33^ and the conveyor effect of waterflow, we show how sampling eDNA from streams can allow quantification of the presence/absence and detection probabilities of a landscape's mammalian fauna, even for most species present at low density, aside from felids and canids (Figs. 2, 3). Our data demonstrate that eDNA approaches can inventory a broad diversity of terrestrial mammals in a cost-efficient manner at the landscape scale, despite recent opinions to the contrary^21^. Specifically, an integrative sampling design with large volumes of water collected from a few large streams within a catchment area, offers a more cost-efficient approach to quantifying biodiversity, compared with the sampling of multiple small streams scattered throughout this same area.

eDNA recovered from water samples integrates an occupancy signal of both aquatic and terrestrial organisms, offering a new monitoring tool for conservation^22,34^. Existing studies comparing eDNA from water^17,18^ or from soil^21^ to camera trapping in the same area showed that for a few terrestrial species, their ability to be detected from eDNA was associated with their behaviour. For instance, Sales et al.^17^ showed that water based eDNA metabarcoding provided detection rates comparable or superior to conventional survey methods per unit of survey effort for three species. However, these studies were conducted on a small scale, with small volumes of water filtered and low eDNA and camera trap sampling efforts, which raised questions on the capacity to use eDNA at larger scales^21^. Our study used a multiscale sampling design in a large landscape, with intensive camera trapping (57 camera trap locations) and large volumes of water (~ 3700 L) from numerous streams (42 locations) and showed that eDNA can, in fact, provide a more cost-efficient approach for monitoring mammals over large landscapes.

Conservation requires not only information about species presence/absence, but also requires quantification of the relative abundance of species to detect early trends in population dynamics^35–37^. Here, we show that a relationship exists at the catchment scale between eDNA detection probability and camera trap detection rates of terrestrial mammals. In addition, the strong correlation between the average eDNA detection probability in 2018 and 2019, the consistent relationship between eDNA detection probabilities, and camera trap detection rates in consecutive years suggest that the results are robust over two years of sampling (Fig. 3). However, the relationship between detection rates and true species abundance is unknown and would require further research before stream eDNA detection could be used as a robust indicator of relative abundance for mammal species.

In agreement with recent findings^17,22^, we observed that detection patterns recovered from stream eDNA were associated with certain ecological characteristics of species, such as diet and body size. Using a formal statistical modelling approach (as suggested in Sales et al.^17^), we showed that eDNA detection do correlate with these ecological traits and with local detection rates of species measured by camera-traps (Supplementary Tables 2 and 3). Our results provide a robust framework to better understand and predict species detection patterns using eDNA. Ecological associations of eDNA detection rates were mirrored in our detections by taxonomic groups (Fig. 4), where we observed a higher detection of ungulates, lagomorphs and small mammals, and a poorer detection of carnivores. Species with an animal-based diet (top predators) were much less detectable via eDNA than those with a mixed (omnivorous predators) or exclusively plant-based diet (herbivores). This was true, even when variation in local camera detection rate and the positive effect of animal body size were accounted for. The detection curves in Fig. 4 showed the lowest eDNA detection profiles for strict carnivorous species like felids^29^, and a slightly higher detection profile for mustelids and canids, which included omnivorous species like *Martes americana* for the former, and *Canis familiaris* and *Canis latrans* for the latter^42^. Lower detection rates for felid may stem from species’ ecologies^17^. We found a similar detectability for omnivore and herbivore species (Supplementary Table 3) which could result from a high proportion of plants, seeds and fruits in the diet of omnivore species at the time of the sampling^38^. This, along with a very large body size, likely explains the higher eDNA detection profile of bears as compared to strict carnivore species (Fig. 4). This result suggests that detection of omnivores may vary seasonally in response to changes in their diet. As a result, accurate monitoring of this ecological group might require extra constraints on protocols such as maintaining the same sampling period every year or conducting seasonal sampling (which would increase costs). Despite the lack of statistical evidence for higher detection of small mammals compared to other herbivores (ungulates and lagomorphs) as we hypothesized (Fig. 4, Supplementary Table 2b), our results showed that among twelve taxa detected by eDNA, six had a detection probability close to 1 in the *catchment* design, and six were detected and identified only by eDNA (Fig. 2, Supplementary Table 1).

Although the intensive catchment sampling was not conducted the same year as the spatial sampling, we are confident that differences between the two are not related to a higher number of species available in 2019 than 2018 in the sampling area. As shown on Fig. 2, more species were detected with camera traps in 2018 than in 2019 and the detection rates were very similar for all taxa except mouse spp group (see Figs. 2, 3a, and Supplementary Figure 6). Site-level eDNA detection probabilities are related to three key environmental and sampling factors that are easy to measure: the area of the watershed where the sample was taken, the intensity of the rain prior to the sampling, and the volume of water filtered (Fig. 4). Our results are consistent with the hypothesis that both the volume of water filtered and the duration of the filtration increase the probability of detection of every species independently, hence a higher expected number of species detected per sample (Supplementary Figure 3, Supplementary Tables 2, 3, and 5). Indeed, a larger volume of water increased the chance of capturing the DNA present at low abundance in the body of water, and a longer sampling time is expected to increase the number of direct (e.g. drinking) and indirect (e.g. bird faeces) animal-water interaction events that occur upstream before or during the sampling^20,26,39^. This knowledge can help increase the cost-efficiency of eDNA surveys by informing when, where, and how to prioritize water collection. With careful modelling of detection probabilities, accounting for species ecology, behaviour, and physical traits as well as environmental and sampling factors, eDNA could become a superior cost-efficient technique for quantitative assessment of wildlife populations.

We recognize that the application of eDNA at a landscape scale has some limitations. While increasing the catchment area increases species detection probability, there might be a threshold where the dilution of DNA in a larger volume of water^40,41^ makes it impossible to detect rare species. This would limit our ability to detect rare species across large areas. The results also showed limited power for felids and one canid present at low-density, which suggest that a greater sampling effort and further optimization of the sampling design are required for these taxa. As terrestrial mammals at the top of the food chain, felids and canids occur at lower densities and are naturally more difficult to detect as they must drink from or enter a body of water for eDNA deposition to occur^23–25^. However, the higher detection of these species with soil-based eDNA^21^ suggest that detection might be increased by sampling water only after heavy rains or in early spring when the snow melts and transfers a higher quantity of eDNA fragments to the stream network. The spatial detection model and predictive curves (Fig. 4) provide a useful means to estimate the sampling effort needed to optimize the detection of these species in their landscapes. We also recognize that while eDNA can provide information on a large number of taxa at once it cannot provide the same level of detail on individuals and populations as can several other survey methods. Camera trapping, for instance, allows the identification of individuals for certain species from photos, the application of statistical models to estimate population size and density^42^, and the determination of behavioural states and life history stages of individuals^43^. This enhanced level of information on focal species, required to assess conservation metrics such as minimum viable population and extinction risk^44^, must be weighed against the higher resources required for data collection, processing and analysis, which limits its application to a small number of species. Conversely, with the same sampling and analysis technique, eDNA could potentially accurately measure parameters of occupancy and detection probabilities of hundreds of species, in all taxonomic groups (mammals, amphibians, fish, etc.), and in many compartments of the ecosystem^20^.

Our study demonstrates that eDNA sampling of the stream network integrates information on terrestrial mammal presence and detection frequencies across an entire landscape, providing a significant advantage over traditional methods that require expensive and time-consuming deployment of sampling devices. The eDNA *catchment* sampling has significant potential application in conservation, including (1) collecting cost-effective quantitative baseline information on diverse wildlife species, from small rodents to large ungulates; (2) tracking changes in community composition over time and the detection frequencies of species in relation to environmental changes and human threats; (3) rapidly providing information on the status and possible disruption of ecological functions of entire ecosystems (e.g., measuring prey availability for large carnivores); and (4) cost-efficiently locating rare and nearly extinct species across large landscapes (although rare felids and canids would require optimized field protocols). Our results suggest that the application of optimized eDNA sampling strategies could transform how biodiversity is monitored in large landscapes, providing decision-makers with more comprehensive quantitative biodiversity data and on faster time scales, ultimately improving our ability to safeguard biodiversity.

## Methods

### Study area and general sampling design

We conducted our study in and around the South Chilcotin Mountains provincial park (hereafter, "SCM") in southwestern British Columbia, Canada. The provincial park was established in 2010 and covers 568 km^2^ of forested mountains and alpine terrain. The SCM is notable for its diversity of large wildlife species, including predators such as grizzly bear (*Ursus arctos horribilis)*, wolverine (*Gulo gulo*), and fisher (*Pekania pennanti*), as well as large ungulates such as moose (*Alces alces*), mule deer (*Odocoileus hemionus*), and mountain goat (*Oreamnos americanus*)^45,46^. In addition to the protected area, there is a mix of logging, mining, ranching, tourism, and private land holdings in the region. In order to obtain comparable sampling of mammal species in the SCM we deployed two protocols in parallel—eDNA and camera trapping—over a 1,216 km^2^ area defined by the two main watersheds (Fig. 1). We were primarily interested in the comparative effectiveness of an eDNA “*spatial sampling design”* (i.e., collecting single water samples in many small streams scattered throughout the study area) vs*.* a *“catchment sampling design”* (i.e., collecting many samples of water from two larger creeks that drain the entire study area) for the detection of terrestrial mammals.

### Camera trap sampling design

We used camera traps to sample trails and logging roads used by wildlife. From June 2018 until September 2019, we installed and monitored 57 camera traps spaced evenly across the region using a hexagonal array of 3 km grids, with one camera per grid^31^. This spacing allows for effective sampling of the range of medium- and large-bodied wildlife species using the area^43,47^. Cameras were set out to maximize the detection of medium and large size mammals, and the survey grid was not expected to detect high alpine (*Ovis canadensis*, *Oreamnos americanus, Ochotona princeps*), aquatic (*Neovison vison*, *Lontra canadensis, Castor canadensis*, *Ondatra zibethiucus*), or volant species (bats) to any significant degree or in any relation to their actual abundance in the environment. We identified to species level all pictures containing wildlife observations, including mammals, birds, and amphibians, although only mammal species were considered in this study and some small mammals could not be reliably identified to species from camera trap images. We classified them using the Camelot software package^48^. For the purpose of this study, we only used data from July to September, in both 2018 and 2019, to provide a robust overview of the mammal community present in the area during the snow-free summer months, and also to get some insight into the relative abundance and distribution of each species. Despite some variation noticeable in some species, the weekly number of camera trap detections was stable over the 3-month sampling period (Supplementary Figure 4).

### eDNA sampling design

Using a Digital Elevation Model and derived hydrology network, we mapped all streams and associated watersheds within the study area. Based on this map and the camera trap locations, we implemented two different sampling designs. In 2018, the eDNA sampling sites were selected to match the spatial distribution of camera trap sites as closely as possible (*"spatial design")*. To do this, we identified the nearest streams to each camera trap and chose an eDNA sampling site on an accessible portion of the stream, wider than one meter, and for which the upstream catchment area contained the camera trap in question. Between September 14^th^ and September 25^th^ 2018 we collected a total of 50 eDNA samples from 42 sites, with two replicates taken at eight of these sites (Fig. 1). The distance between the sampling site and nearest camera trap ranged from 119 to 1666 m. Each sample consisted of the in situ filtration of 30 L of water using a portable Vampire pump on speed level 2 running for 30 min, a sterile filtration kit, and a VigiDNA filter (SPYGEN, Le Bourget du Lac). When possible, water was pumped from the middle of the stream, or otherwise from the fastest flowing portion of the stream accessible from the shore. The total quantity of water that could be filtered depended on the concentration of suspended sediment (CSS). Clogging from sediment sometimes resulted in filtration ending before 30 min; when this happened, we estimated the approximate volume of water filtered based on average flow rates of one litre per minute.

In 2019, we tested a different sampling approach, collecting water only from the mouth of two large creeks that drained the two main watersheds of the study area (*"catchment design"*; Fig. 1). For this protocol we used a 12 Volt Athena Peristaltic Pump (#ATHPERI-10000) that allowed the filtration of a larger volume of water per sample. We used two pumps simultaneously to collect 36 samples over 8 days (4 days per site) between September 10th and 20th 2019. On the first two days, we collected six samples at each site, with the speed of the pump adjusted appropriately to filter: (a) 70–80 L of water over 5 h, (b) 50–60 L over 2 h, and (c) 25–35 L over 30 min (two replicate samples were collected on each setting). Every other day, we collected four samples daily with the pump adjusted to scenario (a). Measurements of the flow were performed every 30 min while sampling and values were averaged over the sampling period to build the covariate. For both years, immediately after the filtration, the filters were filled with 80 mL of CL1 preservative buffer (SPYGEN), labelled, and stored at room temperature.

### eDNA laboratory methods

The DNA extraction was performed in a dedicated controlled DNA laboratory equipped with separate cleanrooms with positive air pressure, UV treatment and frequent air renewal. Decontamination procedures were conducted before and after all manipulations. The DNA extraction and amplification were performed following the protocol of Pont et al.^27^. The amplification step was conducted following Valentini et al.^49^. A universal mammal 12S mitochondrial rDNA primer (Mamm01: 5′-CCG CCC GTC ACY CTC CT-3′ and 5′-GTA YRC TTA CCW TGT TAC GAC-3′; Taberlet et al.^50^) with human blocking primer (5′-CCT CCT CAA GTA TAC TTC AAA GGA CAT TTA ACT-3′) was used to amplify metabarcoding sequences. Five negative extraction controls and two negative PCR controls (ultrapure water) were amplified and sequenced in parallel to the samples to monitor possible contaminations. The purified PCR products were pooled in equal volumes to achieve a theoretical sequencing depth of 300,000 reads per sample. Two libraries were prepared using the MetaFast protocol and two paired-end sequencing (2 × 125 bp) was carried out using an Illumina HiSeq 2500 sequencer on a HiSeq Rapid Flow Cell v2 using the HiSeq Rapid SBS Kit v2 (Illumina, San Diego, CA, USA) for 2018 samples and a MiSeq (2 × 125 bp, Illumina, San Diego, CA, USA) with the MiSeq Flow Cell Kit Version3 (Illumina, San Diego, CA, USA) for 2019 samples following the manufacturer’s instructions at Fasteris (Geneva, Switzerland). Bioinformatic analysis was performed using the programs in the OBITools package (http://metabarcoding.org/obitools, ref.^51^) following Pont et al.^27^. The program ecotag was used for the taxonomic assignment of Molecular Operational Taxonomic Units (MOTUs) with the sequences extracted from release 138 of European Nucleotide Archive (ENA) database (standard sequences) and a curated database, built from the previous one, by retrieving only the mammalians species present in British Columbia (87 species in total) following the IUCN red list (https://www.iucnredlist.org). All MOTUs present in the negative controls were deleted from the database as suggested by Barba et al.^52^. The species found in the negative controls were human and domestic animals: chicken, turkey, pig, dog and cat. These two latter species had different sequences than those present in the field samples. MOTUs showing less than 96% similarity to the reference databases were removed. MOTUs showing a match with sequences of a unique taxa were assigned to the species level. MOTUs showing a match with more than one taxon in the reference database, were assigned to the genus or subfamily taxonomic level. A species of the genus *Marmota* was included in the results despite a match lower than 95%, because the closest taxa available in the database was a close relative species of the same genus. Finally, considering the erroneous assignments of a few sequences to the wrong sample due to tag-jumps^53^, all sequences with a frequency of occurrence below 0.001 per taxon and per library were discarded. After the bioinformatic filters, no reads were found in the extraction and PCR controls. The results were analysed as 1/0 (detection/non-detection) in each eDNA sample (see below).

### Estimating average detection probabilities by species and sampling method

In order to compare the relative detection power of eDNA vs camera trapping, and among eDNA sampling strategies, we estimated for each species (or higher-level taxa when species level identification was not possible), the detection probability per 60 L of water sample for eDNA, and per 60 days of camera trapping. This choice is arbitrary but consistent with the effort level recommended in camera trapping surveys for community assessments^54^. Although no guideline exists for water eDNA survey of terrestrial mammals, 60 L is the volume of water recommended for surveys of aquatic organisms^32^. In this analysis, because we were interested in the global pattern of detection at the level of the study area, we assumed the likelihood of detecting a given species to be equivalent across all sites or samples. We used a Bayesian statistical analysis approach to estimate the detection probabilities of each species with each method and sampling design. All the analyses were performed using program R v4.0.3^55^ and JAGS software^56^ to use Markov Chain Monte Carlo (MCMC) to approximate posterior distributions for every parameter. The detailed description of the approach and models are provided as Supplementary Methods (Supplementary Method 1), while the data and code can be found at the link provided in the Data and Code Availability sections below.

### Sampling costs and cost-efficiency analysis

To obtain the costs per camera trap site and eDNA sample, we estimated the total cost of each survey and then divided it by the number of sites or samples (Table 1, Supplementary Table 4). We considered four budget components: equipment, local transportation, staff time, and lab work. Equipment was divided into reusable items (camera traps, water pumps and rechargeable batteries) that can be used during several survey seasons, and single use items (AA batteries, eDNA sampling kits). Staff time included the time spent by researchers in the field for each sampling scheme. Local transportation included only expenses related to car usage to reach the field sites. It was estimated using the total distance travelled from the field station headquarters to the sites or cluster of sites, and a standard cost per kilometre was applied. Lab work included all activities that cannot be conducted in the field such as eDNA analysis and post-processing of camera trap images for species identification. Lab costs for eDNA included analyses such as DNA extraction, PCR amplification, sequencing, and bioinformatics. The estimated costs were based on total labour days for each technique.

Using results from all eDNA samples and camera traps, we computed the mean site-level diversity, proportion of species turnover between sites^57^ and total diversity or observed species richness (e.g. ref.^58,59^) for each sampling method and design. To compare the effectiveness of each sampling design to recover the diversity of mammals at multiple scales, we used bootstrap resampling method (with 1000 iterations) to estimate species turnover between site and total diversity values and associated 95% confidence intervals along the full gradient of sampling effort allocated in the field (i.e. 1 to 57 camera traps, 1 to 50 eDNA samples in 2018, and 1 to 36 eDNA samples in 2019). Two versions of diversity parameters were computed for eDNA: one that considered all the taxa detected by this method, the other that included only species detectable and identifiable by camera trapping methods (i.e., excluding volant mammals, most species of small rodents, and semi-aquatic species). Finally, for each method and year, we multiplied the number of samples or sites by their respective costs (Table 1) to plot the relationship between each diversity parameter and the corresponding cost of deploying *N* camera traps or collecting and analysing *N* water samples.

### Modelling site diversity (number of species per sample) and species detection probabilities in relation to environmental factors, local abundance, and taxonomic group

First, we used a Generalized Linear Model (GLM) to analyse the effects of environmental factors and sampling covariates on the number of species detected by eDNA at the site level. We used Poisson regression, which assumes that the outcome variable comes from a Poisson distribution and uses the logarithm as the link function. Our analysis was conducted on eDNA samples from both 2018 and 2019 with all taxa detected by this method (Fig. 2). We considered four covariates: area of the upstream watershed, volume of water filtered, rainfall on the day preceding the sampling (www.worldweatheronline.com/gold-bridge-weather-history), and year (as factor) to account for possible annual variation not explained by the three other covariates. For each set of models, we tested all possible combinations of one, two, three, and four covariates in an additive manner.

Second, we used a similar GLM approach to analyse the spatial relationship between eDNA detection of a species at a site and its average detection rate across all camera traps located in the catchment defined by the eDNA sampling site. Detection rates contain information about local animal density and intensity of their local activity, and was not used in our study as a proxy for species relative abundance as the relationship between animal density and detection rates is complex and species dependent (see for instance ref.^60–62^). We used a Binomial logistic regression, which assumes that the outcome variable comes from a Binomial distribution with a logit link. In addition to the local detection rate, we also considered the environmental and sampling factors described above, and a family-level taxonomic effect in one set of models (see Supplementary Table 2a), or, in a second set of models, the diet and average weight of the species^63^ instead of the taxonomic group (see Supplementary Tables 3a,b). For each set of models, we tested all possible combinations of one, two, three, four, five, six, and seven covariates in an additive manner. For this analysis, we only considered species that were targeted by our camera trap survey (see above). We used a model selection approach based on Akaike Information Criteria for small sample size (*AICc*) to compare models with each other and with the null model^64^. The model with the smallest *AICc* was considered to best describe the data. Competing models with *AICc* differences lower than 2 were considered equivalent. Finally, we used the parameter estimates from the best model to predict the number of species detected and the species detection probabilities per sample under different plausible sets of values for all significant explanatory variables.

## Supplementary Information

Supplementary Information.

## Data Availability

All Illumina raw sequences data are available on Dryad at with the identifier https://datadryad.org/stash/share/29rN8nHrTQpt3l3wuUuDil4ebW2AIQcH13Ll43PQ2lg.

## Code availability

The camera trapping datasets that support the findings of this study and R code used to produce the results are provide R codes in GitHub at 10.5281/zenodo.4771193.
