# Supplementary material for: eDNA sampled from stream networks correlates with camera trap detection rates of terrestrial mammals
Source: Sci Rep. 2021 Jun 15;11:11362. doi: 10.1038/s41598-021-90598-5 (PMC8206079; doi:10.1038/s41598-021-90598-5)
Supplement: Supplementary file 1 — Supplementary Information. [file 41598_2021_90598_MOESM1_ESM.docx]

Manuscript title: eDNA sampled from stream networks correlates with camera trap detection rates of terrestrial mammals

SUPPLEMENTARY FIGURES

**Supplementary Fig. 1 |** General geographic location of the study area in British Columbia, Canada. The map was created using ArcGIS Desktop v10.7 (https://www.esri.com/).


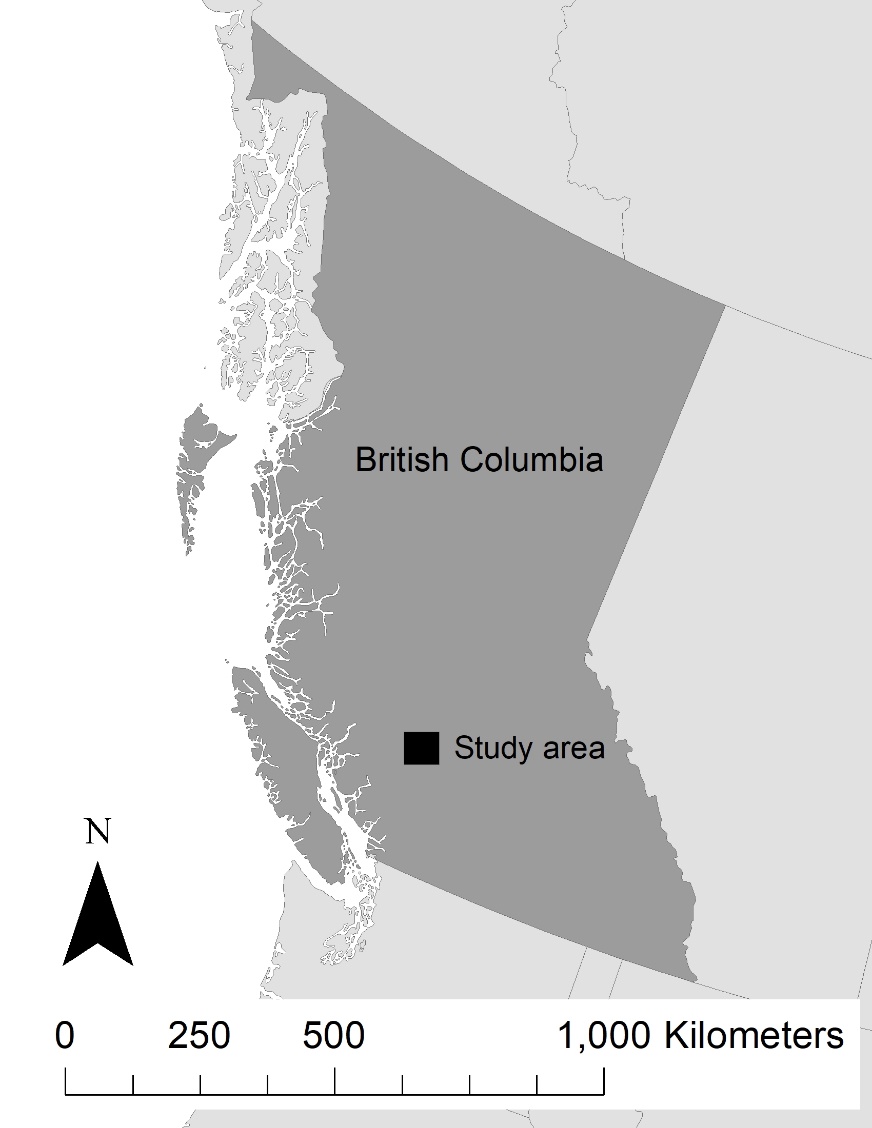


**Supplementary Fig. 2 |** Camera trap (left) *vs.* eDNA (right) species detection maps within the study area. The top of the figure indicates North and the scale is given by the latitude and longitude graduations shown in Universal Transverse Mercator (UTM) in meters (see supplementary Fig. 1 for the general location of the study area in British Columbia). For the camera trap surveys, the size of the circle indicates the average number of independent detections per day at the camera trap station. For eDNA sampling, the circle only indicates that the species was detected in at least one sample at the sampling location. The main streams are shown on the maps (blue lines) as well as the main Land Cover types. Code descriptions: 1,2,5,6 = forest; 8, 11 = grasslands and 10, 12 = shrublands; 14 = wetland, 16 = barren lands; 17 = urban; 18 = water and 19 = snow and ice. The UTM coordinates in meter are shown on both x and y axis. The maps were created using R v4.0.3 (2020)(R Core Team, 2020) (https://cran.r-project.org/).


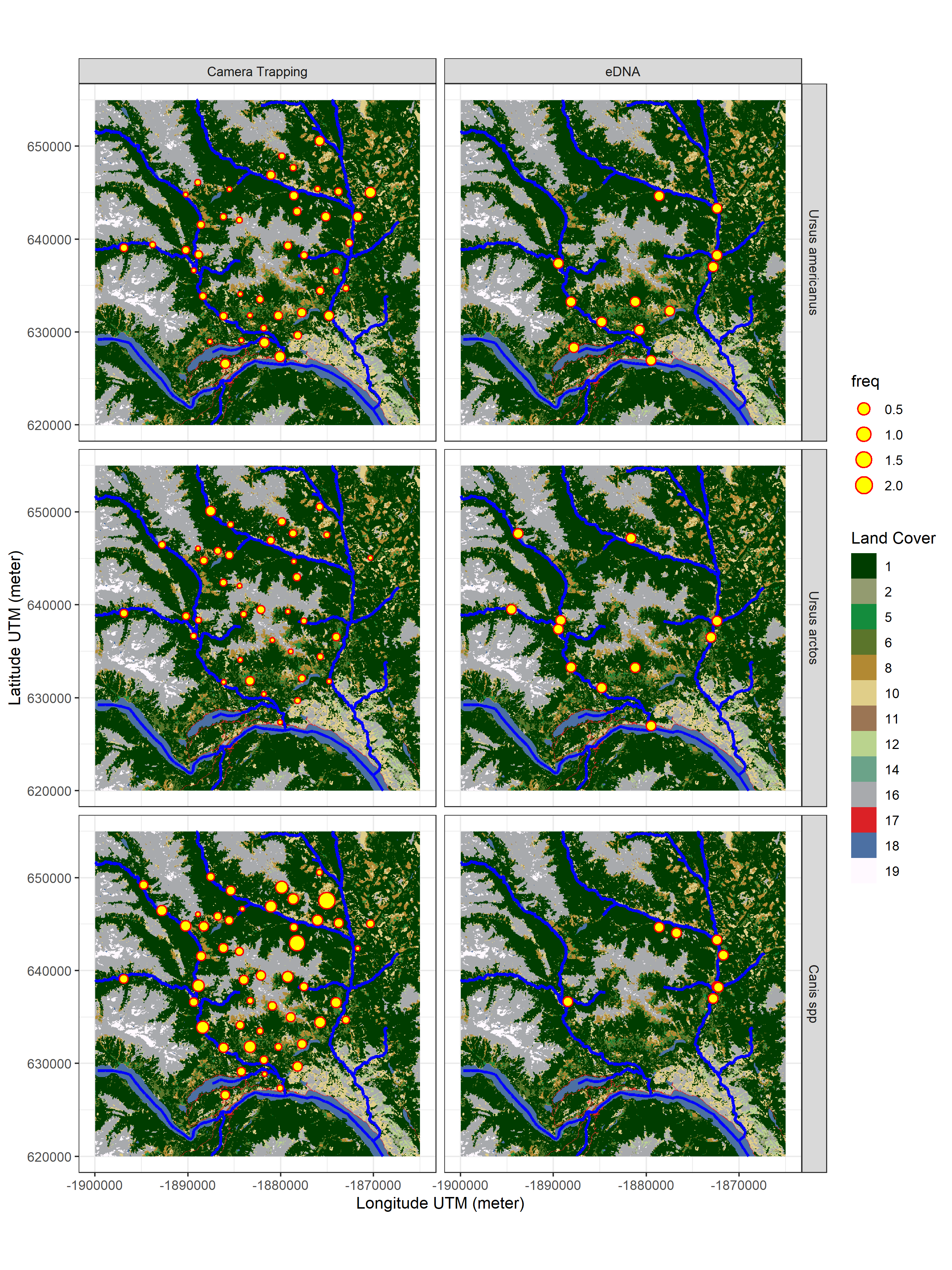


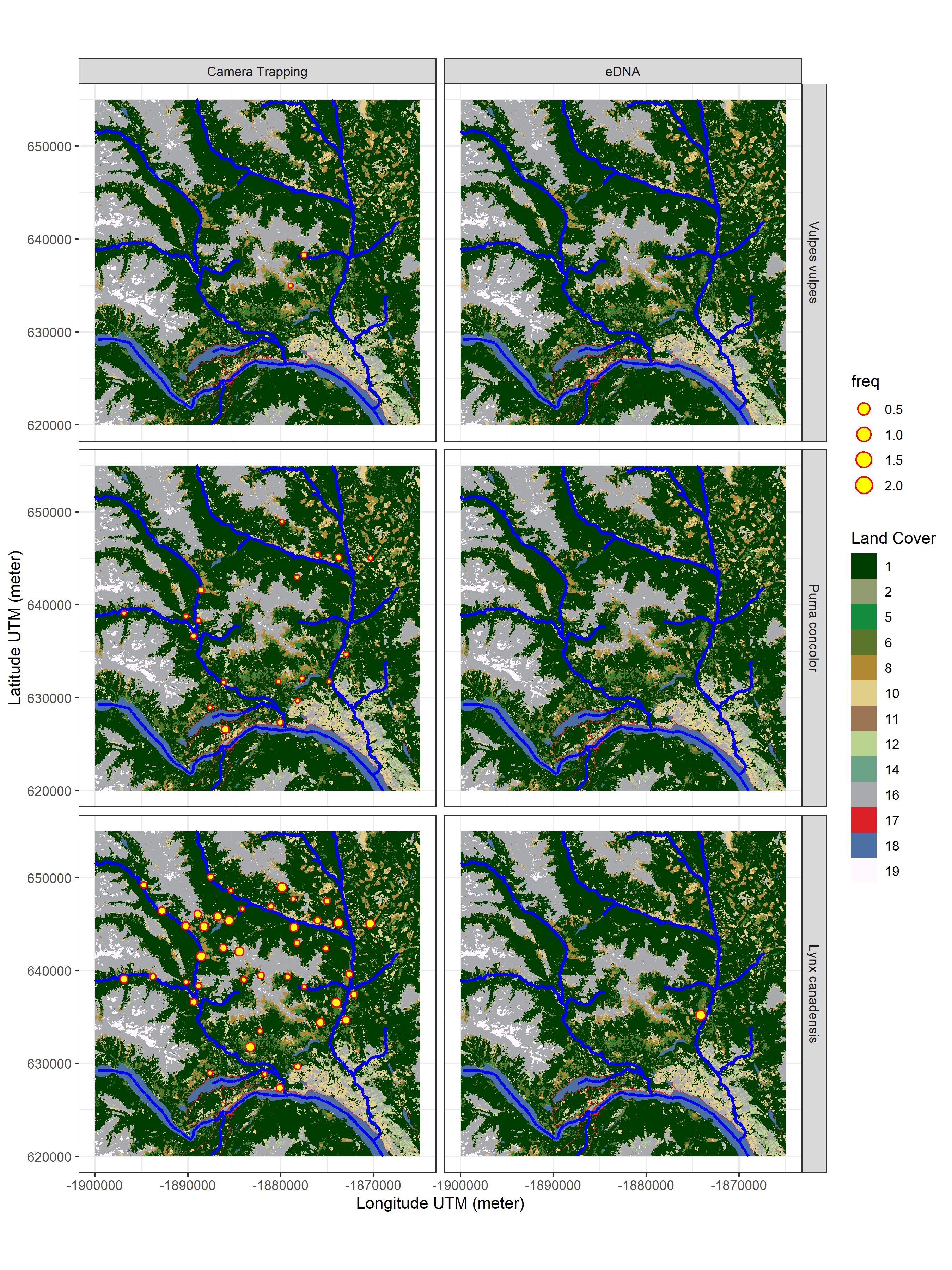


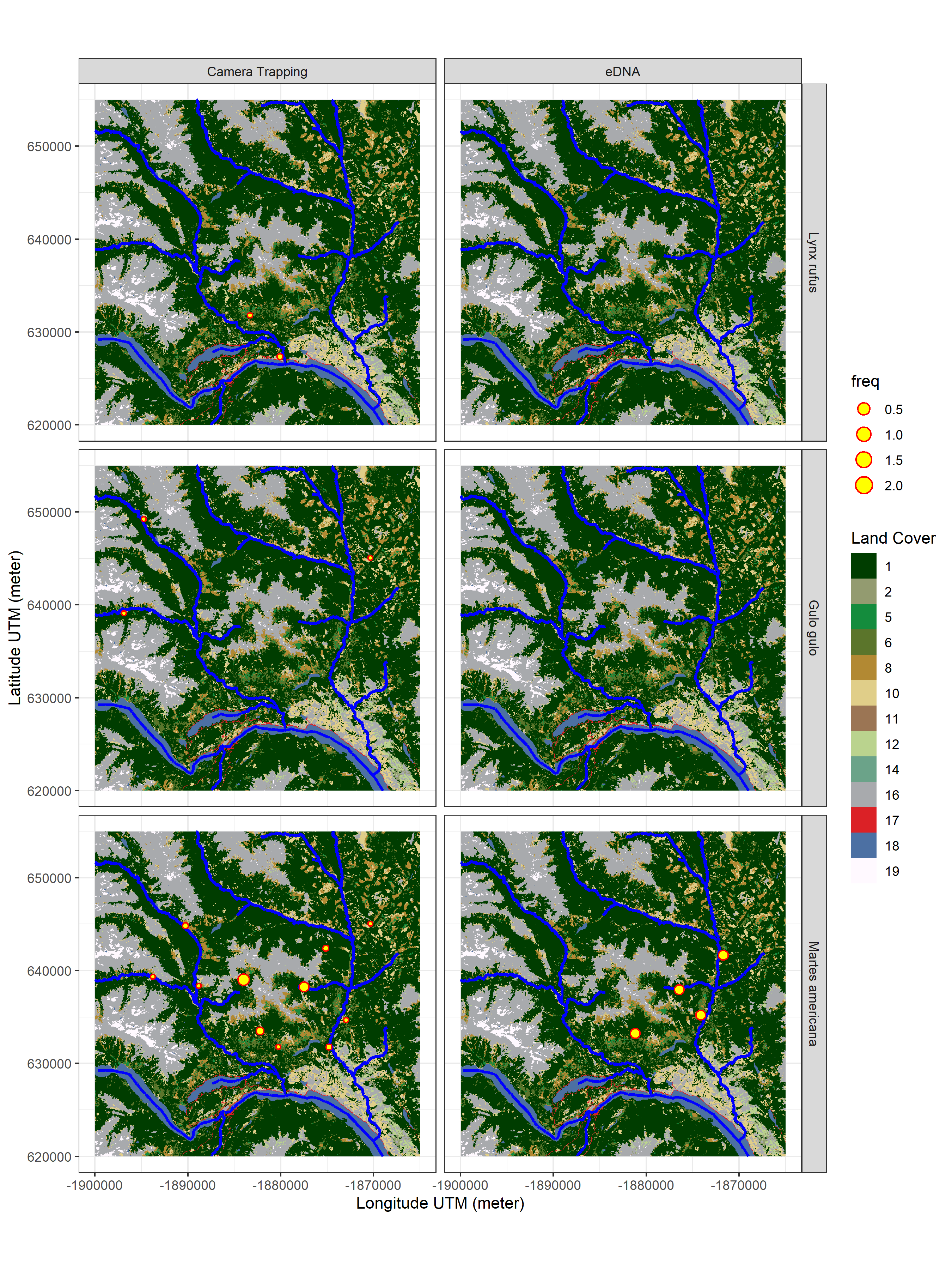


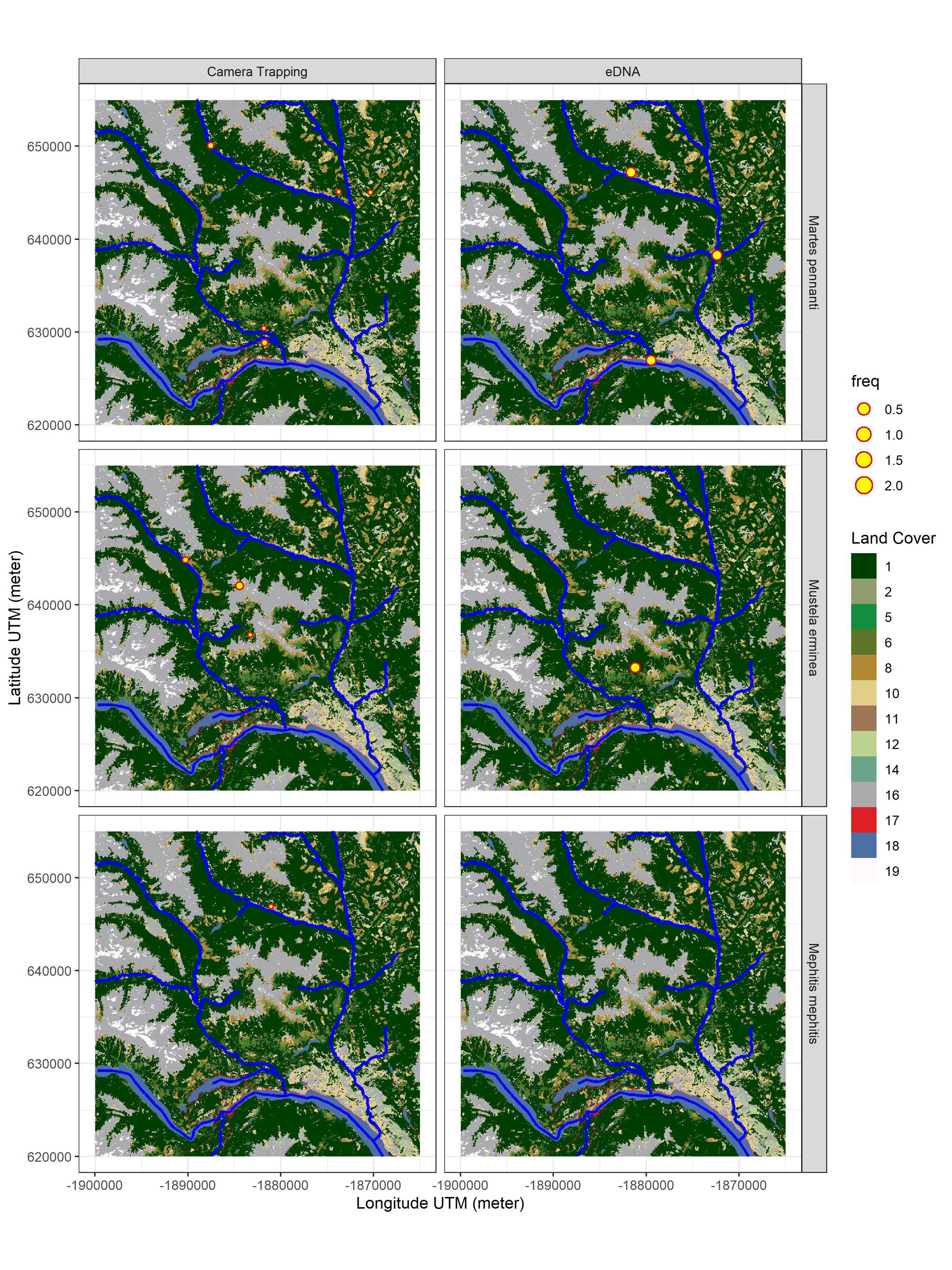


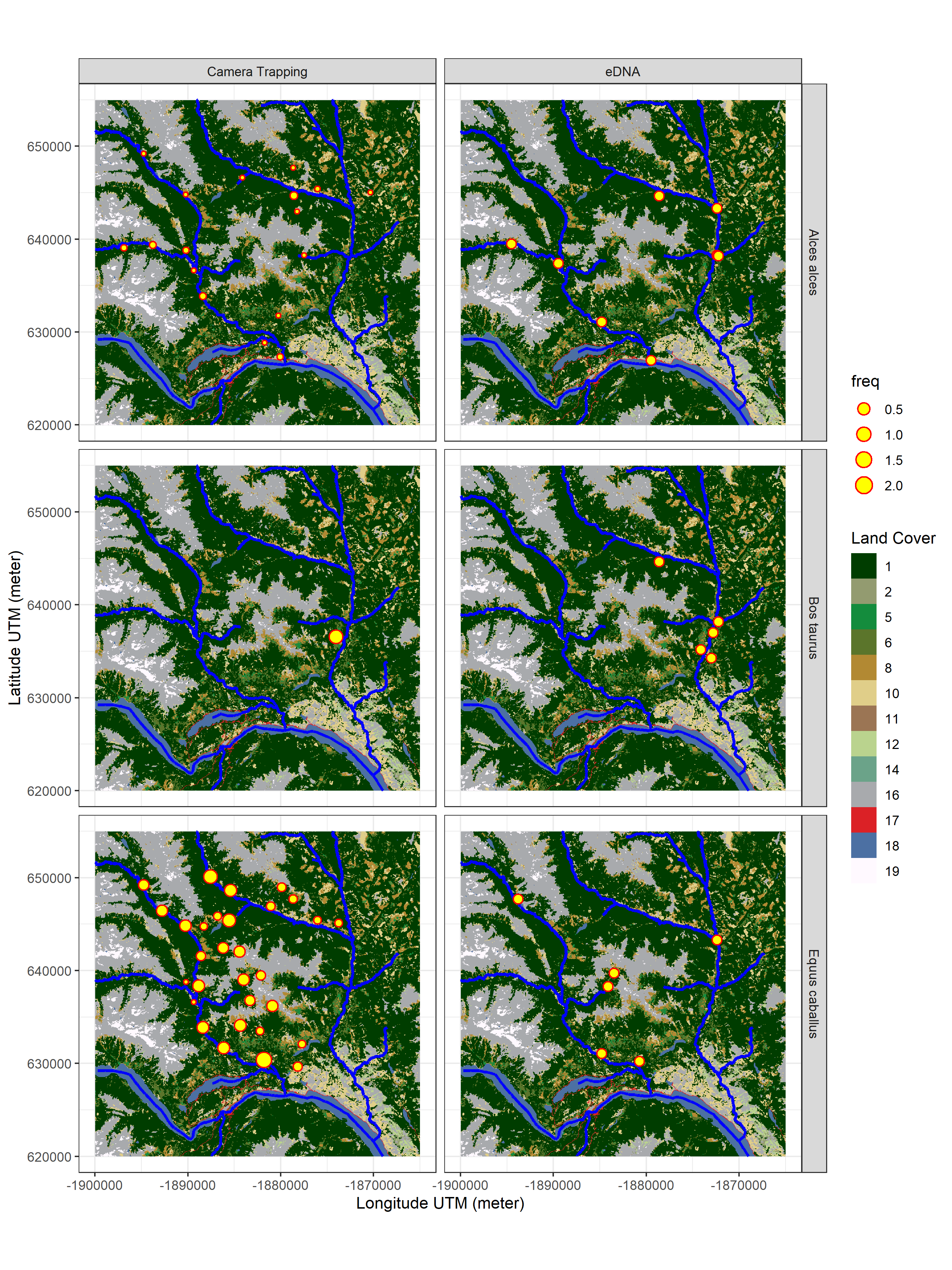


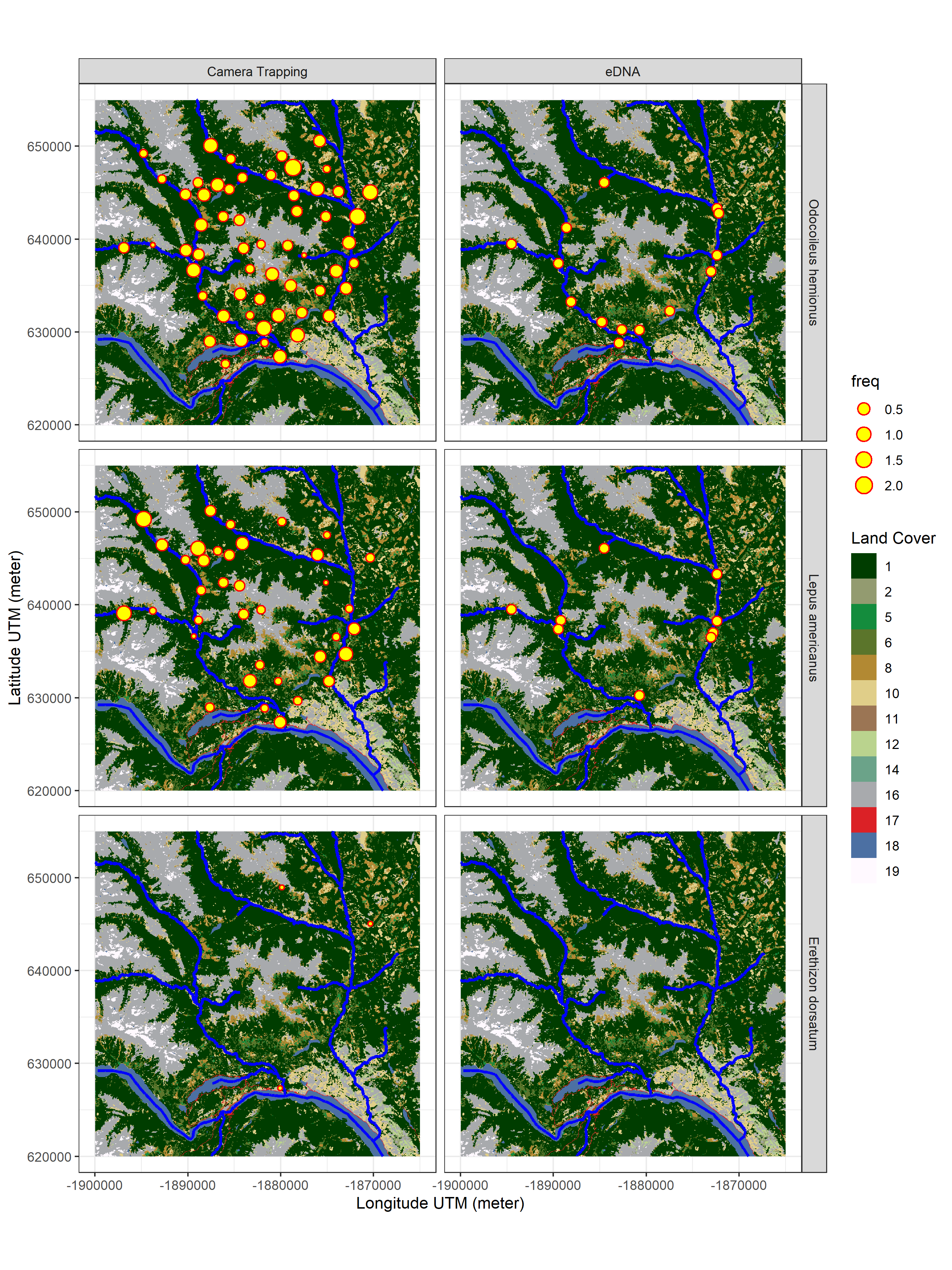


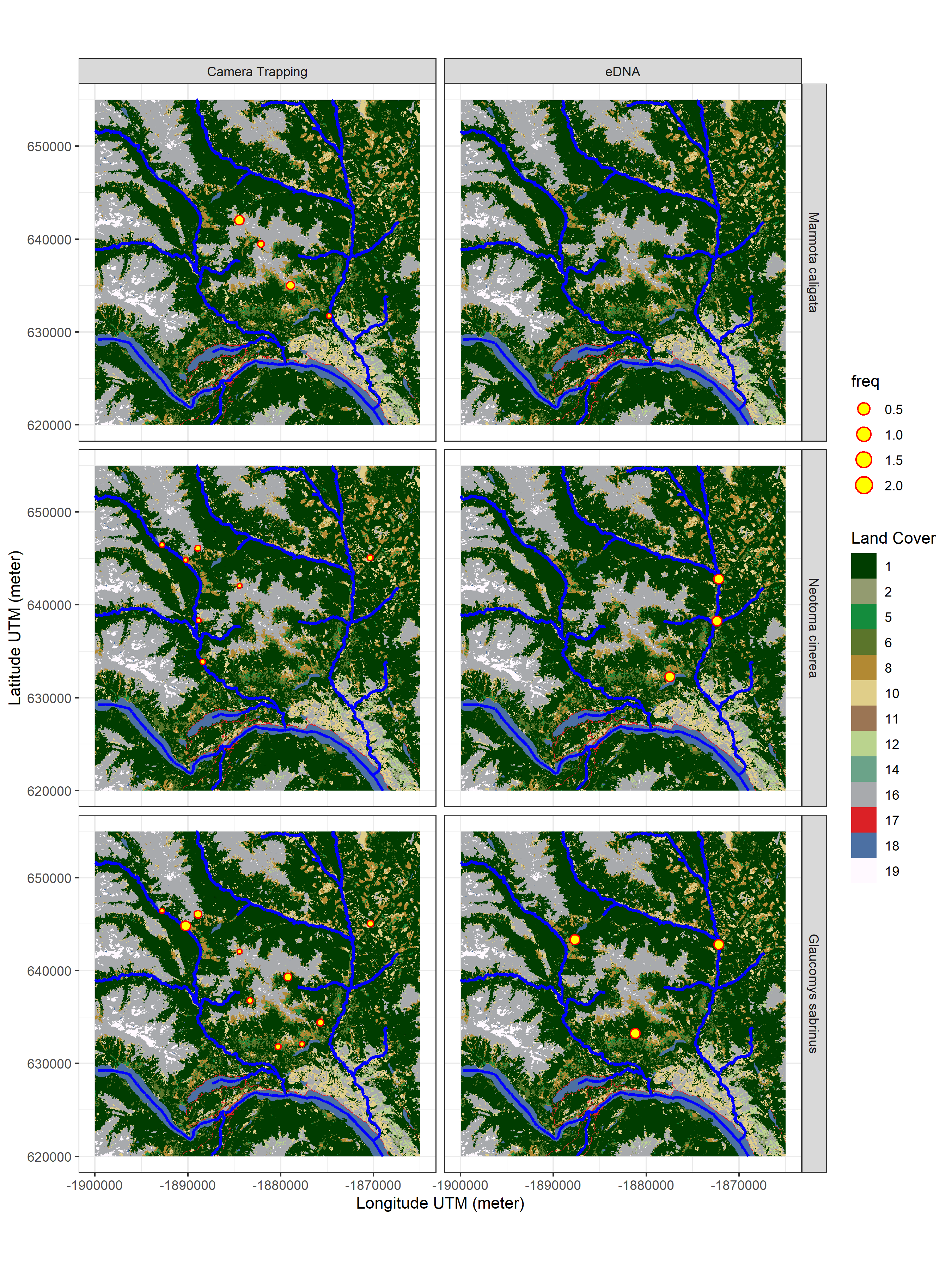


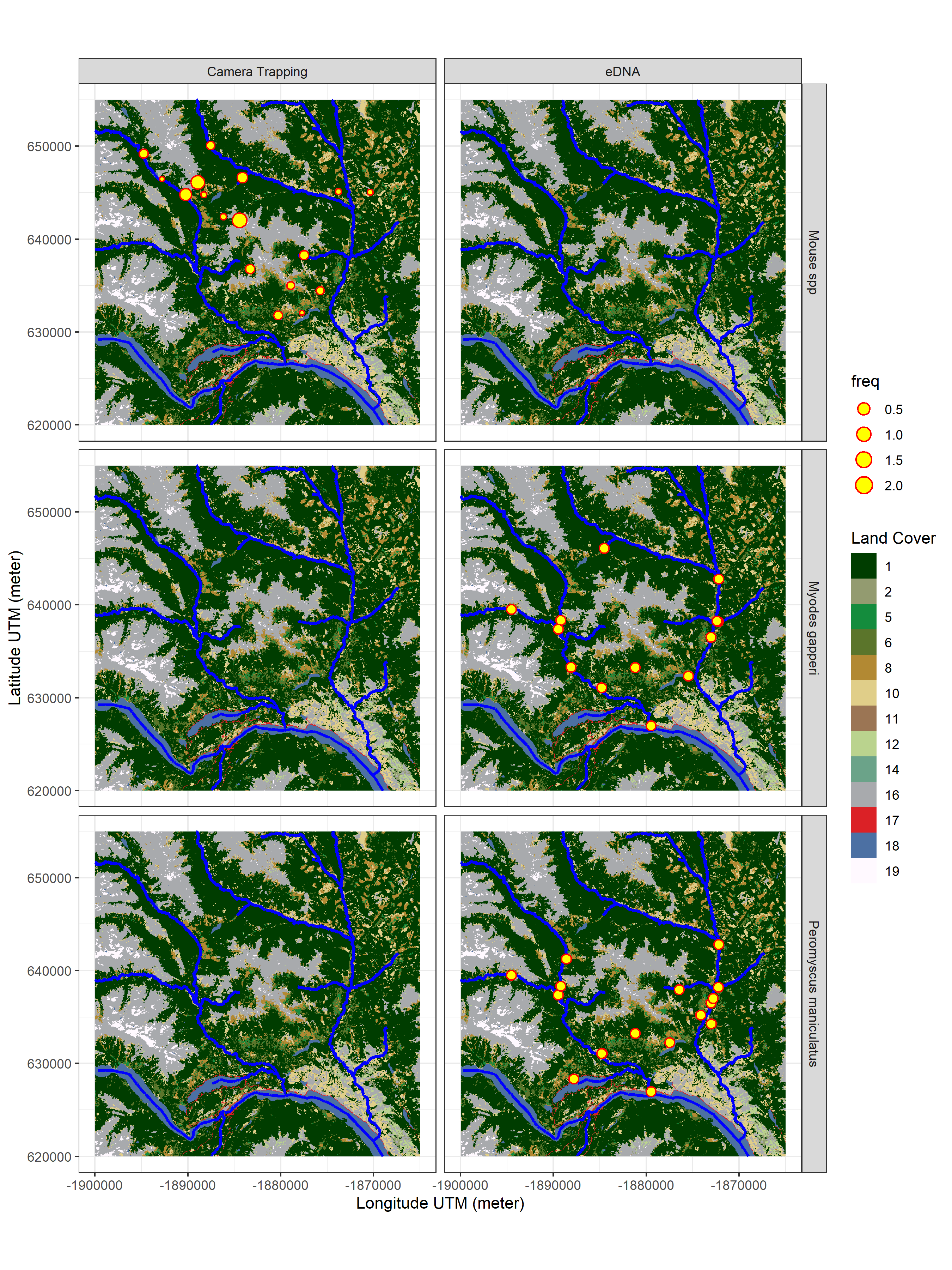


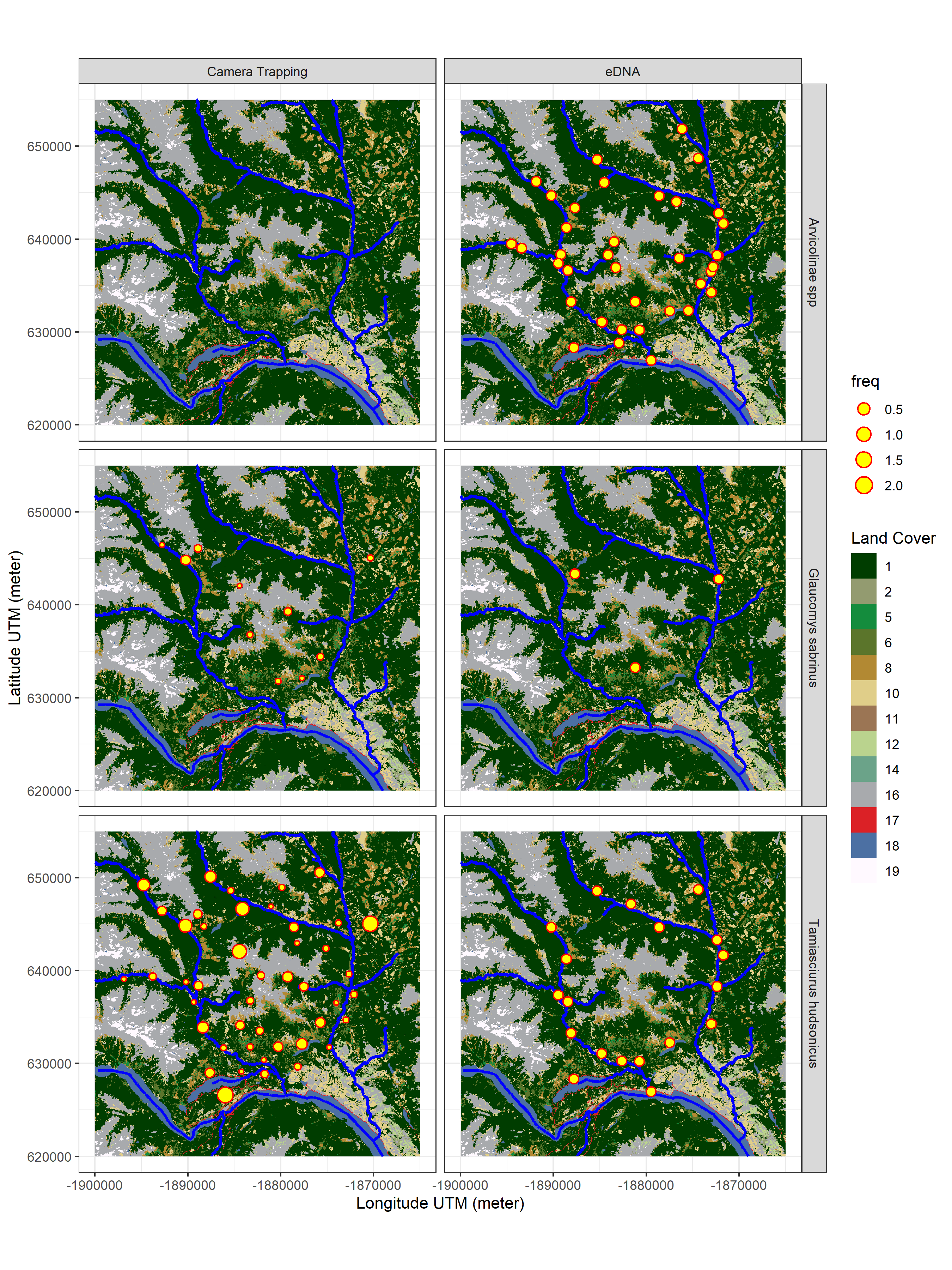


**Supplementary Fig. 3 |** Total species diversity detected per sample in relation to environmental and sampling factors. The curves below show the relationship between the number of species detected per sample (y-axis), the area of the catchment (x-axis) drained by the stream where the sample was collected, the rain the day prior to sampling (blue for heavy rain, red for no rain), and the volume of water filtered (30, 60 and 90 litres from left panel to right panel). Predictions are based on the parameter estimates of the best model described in Supplementary Tables 5a and 5b. The solid lines represent the mean predicted value and the grey areas show the Standard Error associated with the prediction. These results show that the cost-efficiency of eDNA sampling could be further improved by collecting water samples after heavy rainfall. The model predicts that the filtration of 90 litres of water would yield the detection of 23 species when the filtration occurs after heavy rain (30 mm) and from a stream that drains a 60,000 hectares catchment. Under the same weather conditions, the filtration of 60 litres of water, which is the standard for monitoring of fish species (Cantera et al., 2019; Pont et al., 2018), would yield the detection of only 19 species.


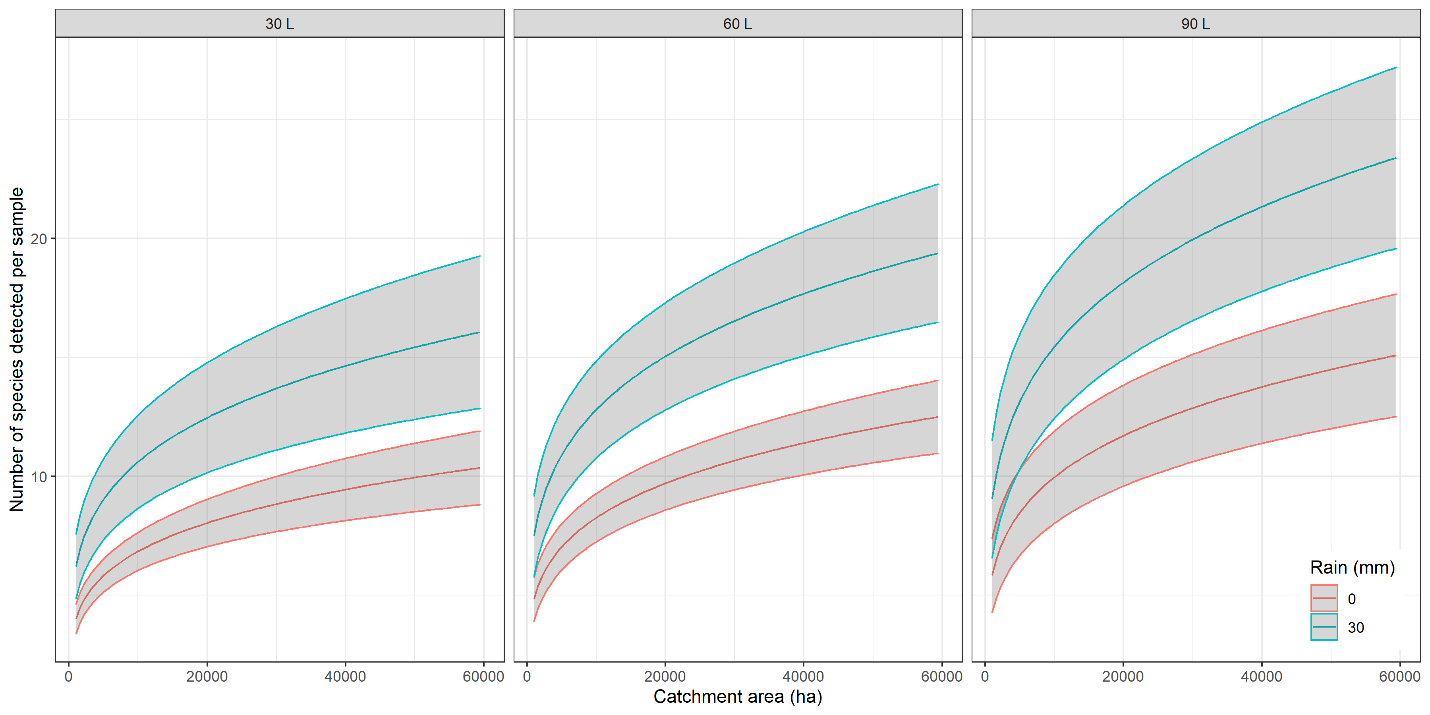


**Supplementary Fig. 4 |** Trends of camera trap detections per species during the three-month survey. The histograms below show the total number of 1-minute independent detections (square transformed, y-axis) per week (y axis) across the camera grid for each species in 2018 (red) and 2019 (blue).


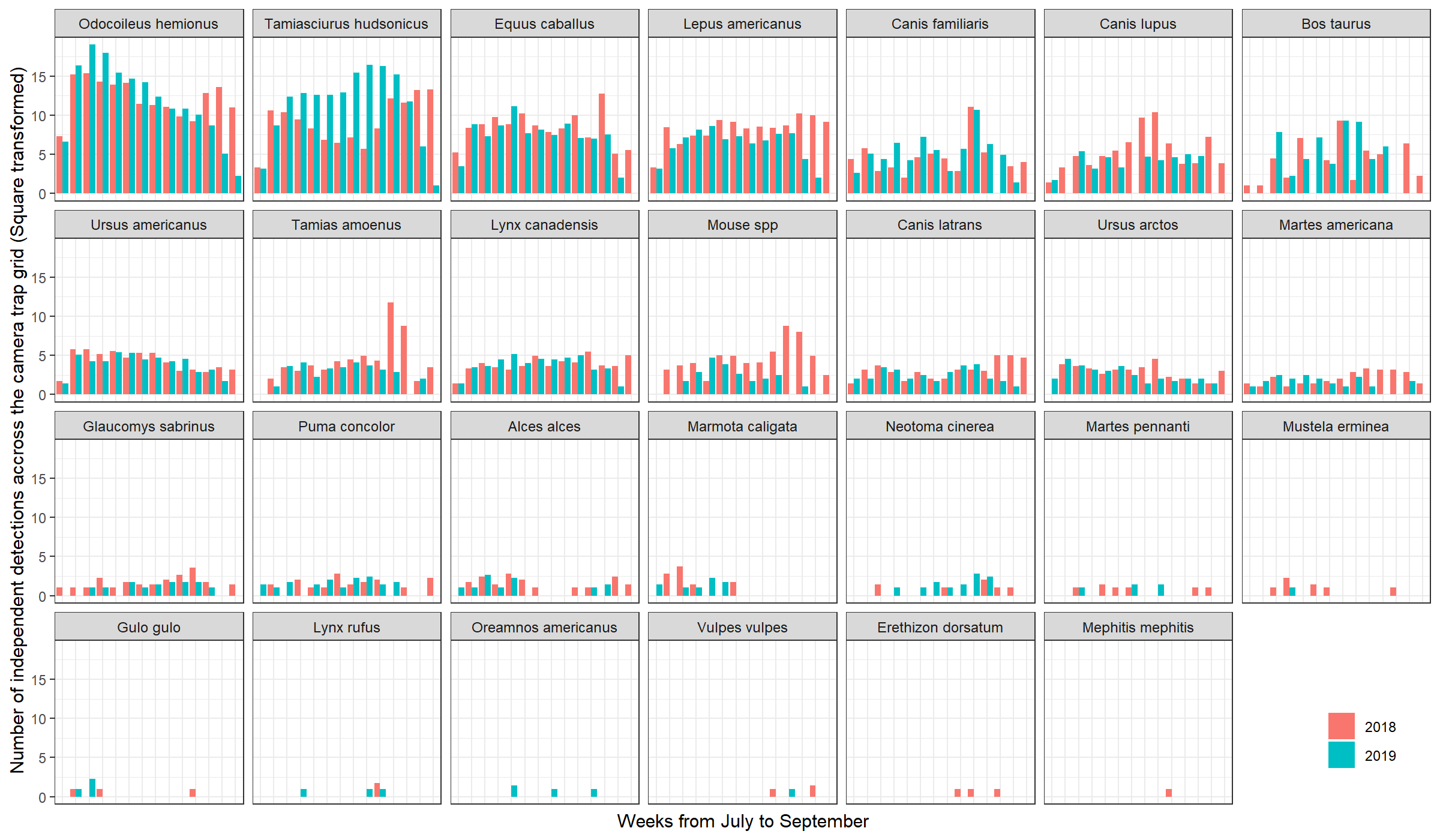


**Supplementary Fig. 5 |** The diagram below shows the number of taxa detected by each method, and by unique combination of 2,3 and 4 methods. For instance the diagram tells that 18 taxa were detected by all 4 methods, 10 taxa were detected only by eDNA methods, 6 by camera trap method only, etc.
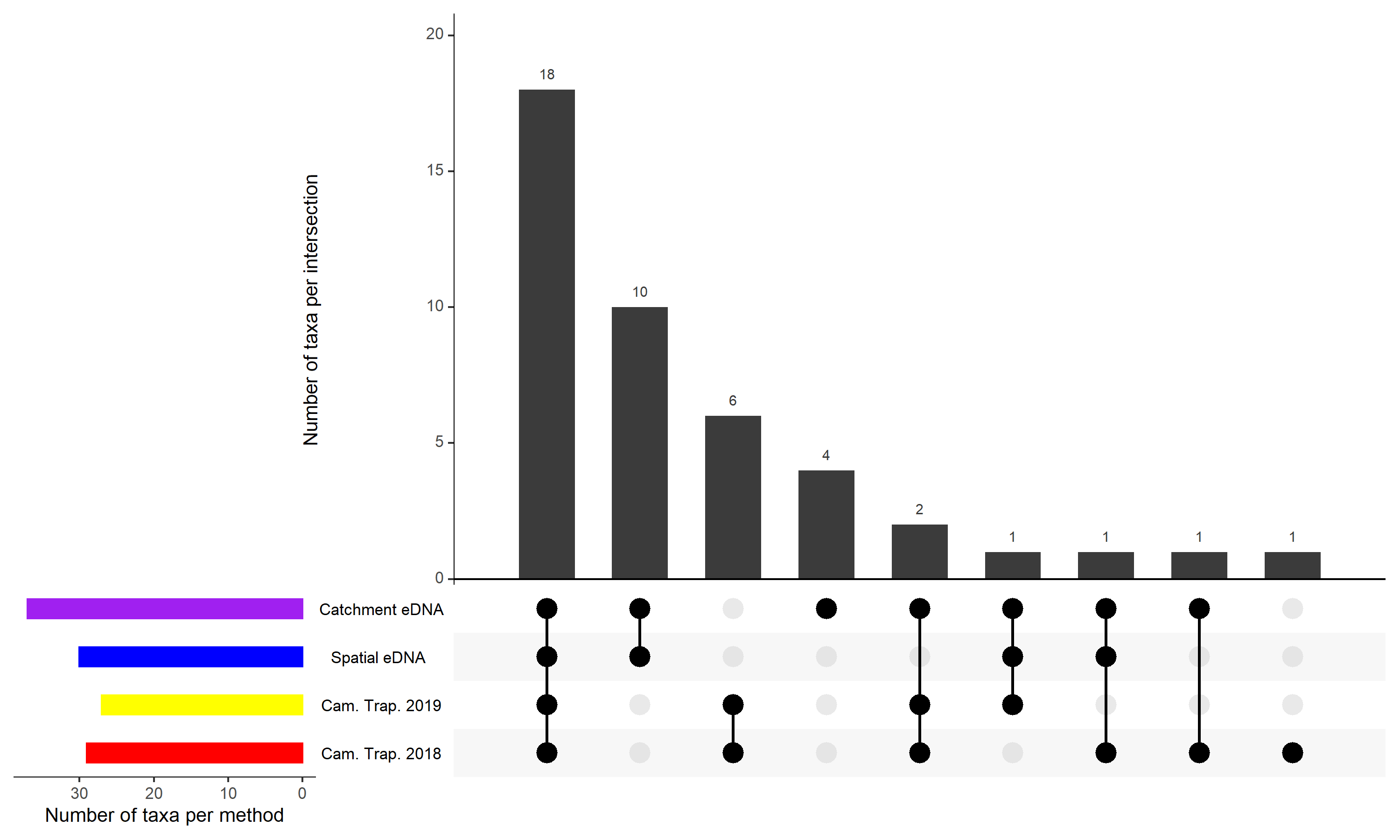


**Supplementary Fig. 6 |** The dots shown on the figure below represent the difference between 2019 and 2018 average detection rates for every taxa detected on the camera trap grid. A negative value indicate a higher detection in 2018 than in 2019. Black dots (N=22) indicate differences smaller than 0.1. Red dots (N=5) indicate differences greater than 0.2.


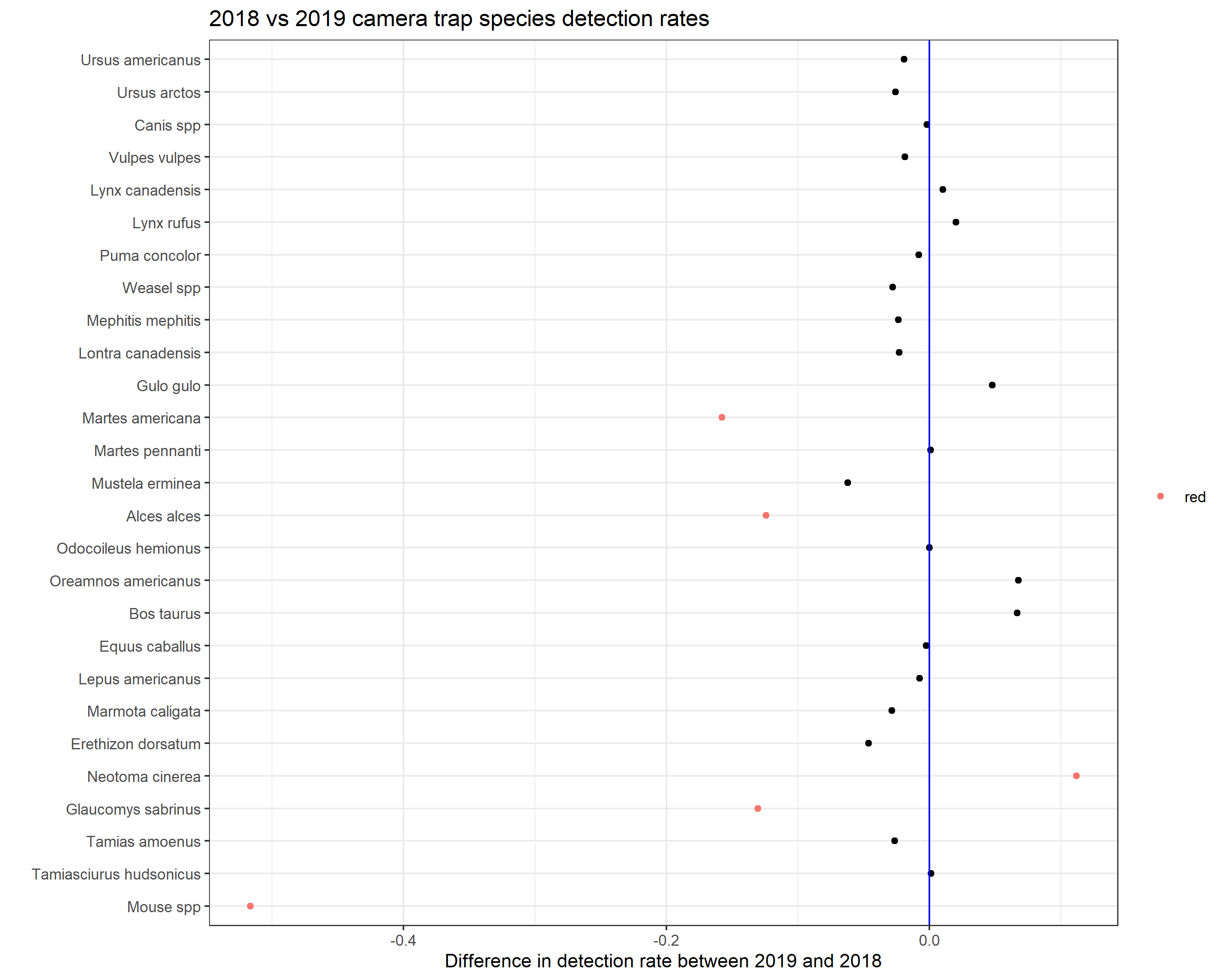


SUPPLEMENTARY TABLES

**Supplementary Table 1a** **|** Raw table of all taxa observed in the eDNA and camera trap surveys. Taxa for which taxonomic adjustments were made as described in the Methods section are identified with a numeric note and explained at the bottom of the table. “*” indicates a possible contamination, e.g., species detected even though evidence suggests it is not present in the study area. See footnotes for most likely source of contamination. The *eDNA REF DB* column corresponds to the number of unique sequences present in the 12S reference database for the taxa listed (see Klinkenberg, Brian. 2019 for the complete list of taxa). *Study Area* column indicates if the species is known to be present in the study area. A sign “-“ stands for not present or not detected, “*X*” for present or detected, “*OS*” for Occasional Sighting, and “*?*” for Presence unknown. *Camera trap* and *eDNA* columns show if the species was detected with the respective method during the course of the project (July-September 2018 and July-September 2019). Camera trapping detected some mustelids and small rodents that could not be identified to the species-level, and are not included in the table below.

| *Family* | *Scientific Name* | *Common Name* | *eDNA REF DB* | *Study Area* | *Camera trap* | *eDNA* |
| --- | --- | --- | --- | --- | --- | --- |
| Bovidae | *Oreamnos americanus* | Mountain Goat | 2 | X | X | X |
|  | *Ovis canadensis* | Bighorn Sheep | 3 | X | - | X |
|  | *Bos taurus* | Cow | 7 | X | X | X |
| Cervidae | *Alces alces* | Moose | 3 | X | X | X |
|  | *Cervus canadensis* | American Elk | 1 | OS | - | - |
|  | *Odocoileus hemionus* | Mule Deer | 1 | X | X | X |
|  | *Rangifer tarandus** | Caribou | 1 | - | - | X |
| Canidae | *Canis latrans* | Coyote | 2 | X | X | - |
|  | *Canis lupus lupus* | Timber Wolf | 21 | X | X | - |
|  | *Canis lupus familiaris* | Dog | 21 | X | X | - |
|  | *Canis spp*^1^ | Undifferentiated canid |  |  |  | X |
|  | *Vulpes vulpes* | Red Fox | 1 | X | X | - |
| Felidae | *Felis catus**^,2^ | Domestic Cat | 3 | X | - | X |
|  | *Lynx canadensis* | Lynx | 1 | X | X | X |
|  | *Lynx rufus* | Bobcat | 2 | X | X | - |
|  | *Puma concolor* | Cougar | 4 | X | X | - |
| Mephitidae | *Mephitis mephitis* | Striped Skunk | 1 | X | X | - |
| Mustelidae | *Gulo gulo* | Wolverine | 2 | X | X | X |
|  | *Lontra canadensis* | River Otter | 1 | X | X | X |
|  | *Martes americana* | American Marten | 1 | X | X | X |
|  | *Mustela erminea* | Short-tailed Weasel | 2 | X | X | X |
|  | *Mustela frenata* | Long-Tailed Weasel | 2 | X | - | X |
|  | *Mustela nivalis* | Least Weasel | 1 | X | - | X |
|  | *Neovison vison* | American Mink | 3 | X | - | X |
|  | *Pekania pennanti* | Fisher | 4 | X | X | X |
| Procyonidae | *Procyon lotor* | Northern Raccoon | 4 | X | - | - |
| Ursidae | *Ursus americanus* | Black Bear | 4 | X | X | X |
|  | *Ursus arctos* | Grizzly Bear | 5 | X | X | X |
| Molossidae | *Nyctinomops macrotis* | Big Free-tailed Bat | 1 | ? | - | - |
| Vespertilionidae | *Corynorhinus townsendii* | Townsend's Big-eared Bat | 1 | X | - | - |
|  | *Eptesicus fuscus* | Big Brown Bat | 2 | X | - | X |
|  | *Euderma maculatum* | Spotted Bat | 1 | ? | - | - |
|  | *Lasionycteris noctivagans* | Silver-Haired Bat | 1 | X | - | X |
|  | *Lasiurus cinereus* | Hoary Bat |  | ? | - | - |
|  | *Myotis californicus* | California Myotis | 2 | ? | - | - |
|  | *Myotis ciliolabrum* | Western Small-Footed Myotis | 2 | ? | - | - |
|  | *Myotis evotis* | Western Long-Eared Myotis | 1 | X | - | - |
|  | *Myotis lucifugus* | Little Brown Myotis | 5 | X | - | - |
|  | *Myotis thysanodes* | Fringed Myostis | 1 | ? | - | - |
|  | *Myotis volans* | Long-Legged Myotis | 1 | X | - | - |
|  | *Myotis yumanensis* | Yuma Myotis | 2 | X | - | - |
|  | *Myotis spp*^3^ | Undifferentiated Myotis species |  |  |  | X |
| Leporidae | *Lepus americanus* | Snowshoe Hare | 1 | X | X | X |
| Ochotonidae | *Ochotona princeps* | Common Pika | 2 | X | - | X |
| Equidae | *Equus caballus* | Horse | 7 | X | X | X |
| Castoridae | *Castor canadensis* | Beaver | 1 | X | - | X |
| Cricetidae | *Lemmus trimucronatus* | Nearctic Brown Lemming | - | ? | - | - |
|  | *Microtus longicaudus* | Long-Tailed Vole | - | X | - | - |
|  | *Microtus montanus* | Montane Vole | - | X | - | - |
|  | *Microtus pennsylvanicus* | Meadow Vole | - | X | - | - |
|  | *Microtus richardsoni* | North American Water Vole | - | ? | - | - |
|  | *Myodes gapperi* | Southern Red-Backed Vole | - | X | - | X |
|  | *Neotoma cinerea* | Bushy-Tailed Woodrat | 2 | X | X | X |
|  | *Ondatra zibethiucus* | Common Muskrat | 1 | X | - | X |
|  | *Peromyscus maniculatus* | North American Deer Mouse | 1 | X | - | X |
|  | *Phenacomys intermedius* | Heather Vole | - | X | - | - |
|  | *Synaptomys borealis* | Northern Bog Lemming | - | X | - | - |
|  | *Arvicolinae*^4^ | Undifferentiated species |  |  |  | X |
| Dipodidae | *Zapus hudsonius* | Meadow Jumping Mouse | - | X | - | - |
|  | *Zapus princeps* | Western Jumping Mouse | - | X | - | - |
| Erethizontidae | *Erethizon dorsatum* | Porcupine | 1 | X | X | X |
| Muridae | *Mus musculus** | House Mouse | 9 | OS | - | X |
|  | *Rattus norvegicus* | Norway Rat | 7 | ? | - | - |
| Sciuridae | *Glaucomys sabrinus*^5^ | Northern Flying Squirrel | - | X | X | X |
|  | *Marmota caligata*^6^ | Hoary Marmot | - | X | - | X |
|  | *Neotamias amoenus* | Yellow-Pine Chipmunk | 2 | X | X | X |
|  | *Tamiasciurus hudsonicus* | Red Squirrel | 1 | X | X | X |
| Soricidae | *Sorex cinereus* | Common Shrew | 1 | X | - | X |
|  | *Sorex hoyi* | Pygmy Shrew | - | X | - | - |
|  | *Sorex merriami* | Merriam's Shrew | - | ? | - | - |
|  | *Sorex monticolus* | Dusky Shrew | - | X | - | - |
|  | *Sorex palustris* | Common Water Shrew | 1 | X | - | - |
|  | *Sorex preblei* | Preble's Shrew | - | ? | - | - |
|  | *Sorex spp*^1^ | Undifferentiated Shrew |  | X | - | X |

* *Rangifer tarandus* is not known in the study area and the nearest known population is more than a hundred miles away. The DNA of this species was however detected in two eDNA samples in 2019 collected the same day in the same river. It is therefore very likely that some DNA of this species has been transferred to the stream above the sampling location shortly before the sample collection. DNA could have been transported by a predator such as *Aquila chrysaetos* or *Canis lupus*, or by a hunter (Coutant et al., 2020). *Mus musculus* and *Felis* spp (most likely *Felis catus*) were only detected once in our eDNA samples. These species are uncommon in the study area their DNA could have been transported on the gear of any human visiting the study area (tourist, fisher, hiker, etc) and subsequently transferred to the stream network.

^1^ In the eDNA samples, we identified 6 unique sequences that can be found in both *Canis lupus lupus* and *Canis lupus familiaris* (5 sequences), or in any of the three *Canis spp* (1 sequence) present in the study area. We decided to keep the assignment of all sequences to the genus level in our analyses.

^2^ The eDNA sequence was matched with the genus *Felis* (1 id value). The sequence was reassigned to *Felis catus* which is the only species of this genus present in the study area.

^3^ In the eDNA samples, we identified 2 unique sequences that were matched with the genus *Myotis* (id=1 and 0.96). These sequences are shared between multiple species and could not be assigned to a particular species.

^4^ Sequences in this group were matched in the reference database with sequences shared between multiple species across multiple genus. All these sequences were therefore assigned to the *Arvicolinae* subfamily.

^5^ *Glaucomys sabrinus* is not present in the DNA reference database. Two unique sequences detected in our samples were matched with *Glaucomys volans* (0.97 and 0.95 id value) which is only present in eastern Canada and were reassigned to *Glaucomys sabrinus*.

^6^ *Marmota canadensis* is the only species of the genus present in the study area but the taxon is not present in the reference database. The unique sequence of the genus *Marmota* that was detected in our samples had a 0.95 id match with the sequence of *Marmota flaviventris*. We therefore decided to attribute the sequence to the *Marmota canadensis* which is the most likely species detected in our eDNA samples.

**Supplementary Table 1b |** Total number of taxa detected by eDNA and camera trapping methods. The figure in each cell represents the number of taxa present in the study area or detected by the method. Number of domestic taxa are shown in parenthesis. These results do not include *Rangifer tarandus*, *Mus musculus* and *Felis* spp*.* whose detection might have resulted from contamination (see Table 1a).

| Order | Present in study area | Detected by eDNA  2018-2019 | | Detected by CT  2018-2019 | |
| --- | --- | --- | --- | --- | --- |
|  |  | Species level | Other level | Species level | Other level |
| Artiodactyla | 5 (1) | 5 (1) ^1^ | - | 4 (1) | - |
| Perissodactyla | 1 (1) | 1 (1) | - | 1 (1) | - |
| Lagomorpha | 2 | 2 | - | 1 | - |
| Carnivora | 19 (2) | 11 | 1 ^2^ | 15 (1) | 0 ^4^ |
| Rodentia | 18 (1) | 10 ^3^ | 1 | 6 | 1 ^4^ |
| Chiroptera | 7 | 2 | 1 | - | - |
| Soricomorpha | 4 | 1 | - | - | - |
| Total | 56 (5) | 32 (2) | 3 | 27 (3) | 1 |

(^1^) *Rangifer tarandus* excluded; (^2^) *Felis catus* excluded; (^3^) *Mus musculus* excluded; (^4^) Camera trapping detected some mustelids that could not be identified to the species-level. We did not include them here as it is not possible to tell if these records include new weasel species not already detected, identified and counted in the Species level column. However, unidentified small rodents detected on camera traps were included in the Other level column as these taxa they most likely belonged to taxa (*Cricetidae* and *Muridae*) distinct from the ones included in the Species level column.

**Supplementary Table 2a |** Modelling stream eDNA species detection in relation to environmental and sampling covariates, and family-level taxonomic group. Results from the generalized linear model selection procedure are shown in the table below, which presents the list of all models compared. For each model, the table shows: *K,* the number of parameters; *AICc,* the value of the Akaike Information Criteria for small samples; *ΔAICc*, the difference AICc value between the best model and the model evaluated; *AICcWt*, the model weight; and *Cum.Wt*, cumulative *AICc* weight. *Model description* indicates which covariates were included in the model: *det.ws* is the average number of detections per species per day, averaged across all camera trap stations present in the catchment, *aws* is the log of the area (km^2^) of the catchment above the sampling site, *vol* is the volume (litre) of water filtered for the sample, *rain* is the precipitation (millimetre) the day prior to the sampling event, and *gp* describes the family-level taxonomic group: *Bears*, *Canids*, *Felids*, *Mustelids*, *Ungulates*, *Lagomorphs*, *Ground rodents*, and *Arboreal rodents*.

| Model description | K | AICc | ΔAICc | AICcWt | Cum.Wt |
| --- | --- | --- | --- | --- | --- |
| det.dna~det.ws+rain+aws+vol+gp | 12 | 1942.94 | 0 | 0.73 | 0.73 |
| det.dna~det.ws+rain+aws+vol+gp+year | 13 | 1944.96 | 2.02 | 0.27 | 1 |
| det.dna~det.ws+rain+aws+gp+year | 12 | 1953.12 | 10.19 | 0 | 1 |
| det.dna~det.ws+rain+aws+gp | 11 | 1961.87 | 18.93 | 0 | 1 |
| det.dna~det.ws+aws+vol+gp | 11 | 1964.2 | 21.27 | 0 | 1 |
| det.dna~det.ws+aws+vol+gp+year | 12 | 1966.22 | 23.28 | 0 | 1 |
| det.dna~det.ws+aws+gp+year | 11 | 1979.26 | 36.33 | 0 | 1 |
| det.dna~det.ws+rain+vol+gp+year | 12 | 1982.81 | 39.88 | 0 | 1 |
| det.dna~det.ws+rain+gp+year | 11 | 1987.64 | 44.71 | 0 | 1 |
| det.dna~rain+aws+vol+gp | 11 | 1993.8 | 50.86 | 0 | 1 |
| det.dna~det.ws+aws+gp | 10 | 1993.89 | 50.95 | 0 | 1 |
| det.dna~rain+aws+vol+gp+year | 12 | 1995.81 | 52.88 | 0 | 1 |
| det.dna~det.ws+rain+vol+gp | 11 | 2003.29 | 60.35 | 0 | 1 |
| det.dna~rain+aws+gp+year | 11 | 2003.99 | 61.05 | 0 | 1 |
| det.dna~det.ws+vol+gp+year | 11 | 2007.71 | 64.78 | 0 | 1 |
| det.dna~aws+vol+gp | 10 | 2010.16 | 67.23 | 0 | 1 |
| det.dna~rain+aws+gp | 10 | 2011.51 | 68.58 | 0 | 1 |
| det.dna~aws+vol+gp+year | 11 | 2012.18 | 69.24 | 0 | 1 |
| det.dna~det.ws+gp+year | 10 | 2017.15 | 74.22 | 0 | 1 |
| det.dna~aws+gp+year | 10 | 2024.61 | 81.67 | 0 | 1 |
| det.dna~det.ws+vol+gp | 10 | 2030.52 | 87.59 | 0 | 1 |
| det.dna~rain+vol+gp+year | 11 | 2035.62 | 92.68 | 0 | 1 |
| det.dna~aws+gp | 9 | 2037.31 | 94.37 | 0 | 1 |
| det.dna~rain+gp+year | 10 | 2040.38 | 97.45 | 0 | 1 |
| det.dna~vol+gp+year | 10 | 2055.1 | 112.17 | 0 | 1 |
| det.dna~rain+vol+gp | 10 | 2056.21 | 113.28 | 0 | 1 |
| det.dna~gp+year | 9 | 2063.89 | 120.95 | 0 | 1 |
| det.dna~vol+gp | 9 | 2077.87 | 134.94 | 0 | 1 |
| det.dna~det.ws+rain+gp | 10 | 2121.51 | 178.57 | 0 | 1 |
| det.dna~rain+gp | 9 | 2174.08 | 231.14 | 0 | 1 |
| det.dna~det.ws+rain+aws+vol | 5 | 2198.23 | 255.29 | 0 | 1 |
| det.dna~det.ws+rain+aws+vol+year | 6 | 2200.24 | 257.3 | 0 | 1 |
| det.dna~det.ws+gp | 9 | 2201.62 | 258.68 | 0 | 1 |
| det.dna~det.ws+rain+aws+year | 5 | 2206.64 | 263.7 | 0 | 1 |
| det.dna~det.ws+rain+aws | 4 | 2213.76 | 270.83 | 0 | 1 |
| det.dna~det.ws+aws+vol | 4 | 2217.37 | 274.43 | 0 | 1 |
| det.dna~det.ws+aws+vol+year | 5 | 2219.37 | 276.44 | 0 | 1 |
| det.dna~det.ws+aws+year | 4 | 2230.34 | 287.4 | 0 | 1 |
| det.dna~det.ws+rain+vol+year | 5 | 2234.46 | 291.53 | 0 | 1 |
| det.dna~det.ws+rain+year | 4 | 2238.01 | 295.08 | 0 | 1 |
| det.dna~det.ws+aws | 3 | 2242.56 | 299.62 | 0 | 1 |
| det.dna~gp | 8 | 2244.8 | 301.86 | 0 | 1 |
| det.dna~det.ws+rain+vol | 4 | 2252.81 | 309.88 | 0 | 1 |
| det.dna~det.ws+vol+year | 4 | 2256.88 | 313.94 | 0 | 1 |
| det.dna~det.ws+year | 3 | 2264.77 | 321.83 | 0 | 1 |
| det.dna~det.ws+vol | 3 | 2277.07 | 334.13 | 0 | 1 |
| det.dna~rain+aws+vol | 4 | 2318.22 | 375.29 | 0 | 1 |
| det.dna~rain+aws+vol+year | 5 | 2320.22 | 377.29 | 0 | 1 |
| det.dna~rain+aws+year | 4 | 2326.44 | 383.51 | 0 | 1 |
| det.dna~aws+vol | 3 | 2331.64 | 388.7 | 0 | 1 |
| det.dna~rain+aws | 3 | 2332.1 | 389.17 | 0 | 1 |
| det.dna~aws+vol+year | 4 | 2333.64 | 390.7 | 0 | 1 |
| det.dna~aws+year | 3 | 2343.63 | 400.69 | 0 | 1 |
| det.dna~aws | 2 | 2353.41 | 410.48 | 0 | 1 |
| det.dna~det.ws+rain | 3 | 2356 | 413.06 | 0 | 1 |
| det.dna~rain+vol+year | 4 | 2356.08 | 413.15 | 0 | 1 |
| det.dna~rain+year | 3 | 2359.41 | 416.48 | 0 | 1 |
| det.dna~vol+year | 3 | 2372.28 | 429.34 | 0 | 1 |
| det.dna~rain+vol | 3 | 2374.52 | 431.59 | 0 | 1 |
| det.dna~year | 2 | 2379.2 | 436.26 | 0 | 1 |
| det.dna~vol | 2 | 2392.39 | 449.45 | 0 | 1 |
| det.dna~det.ws | 2 | 2428.56 | 485.62 | 0 | 1 |
| det.dna~rain | 2 | 2476.1 | 533.16 | 0 | 1 |
| det.dna~1 | 1 | 2537.27 | 594.33 | 0 | 1 |

**Supplementary Table 2b |** The table shows the beta parameter estimates of the best model: det.dna ~ det.ws + rain + aws + vol + gp, see Table 2a.

|  | Estimate | Std. Error | z value | Pr(>\|z\|) |
| --- | --- | --- | --- | --- |
| (Intercept) | -3.513588 | 0.325289 | -10.801 | < 2e-16 *** |
| det.ws | 2.469796 | 0.365415 | 6.759 | 1.39e-11 *** |
| rain | 0.031033 | 0.00637 | 4.872 | 1.10e-06 *** |
| aws | 0.272962 | 0.035406 | 7.709 | 1.26e-14 *** |
| vol | 0.013308 | 0.002944 | 4.52 | 6.19e-06 *** |
| gpCanids | -1.751398 | 0.272528 | -6.426 | 1.31e-10 *** |
| gpFelids | -4.663641 | 0.609111 | -7.656 | 1.91e-14 *** |
| gpMustelids | -2.328098 | 0.223858 | -10.4 | < 2e-16 *** |
| gpUngulates | -0.751969 | 0.256667 | -2.93 | 0.00339 ** |
| gpLagomorphs | -0.512951 | 0.298166 | -1.72 | 0.08537 . |
| gpGround rodents | -0.89882 | 0.192417 | -4.671 | 2.99e-06 *** |
| gpArboreal rodents | -1.012651 | 0.259063 | -3.909 | 9.27e-05 *** |

Signif. codes: 0 ‘***’ 0.001 ‘**’ 0.01 ‘*’ 0.05 ‘.’ 0.1 ‘ ’ 1

**Supplementary Table 2c |** The table shows the beta parameter estimates of the best model that includes the year effect: det.dna ~ det.ws + rain + aws + vol + gp + year, see Table 2a.

|  | Estimate | Std. Error | z value | Pr(>\|z\|) |
| --- | --- | --- | --- | --- |
| (Intercept) | -3.501466 | 0.412004 | -8.499 | < 2e-16 *** |
| det.ws | 2.470048 | 0.365462 | 6.759 | 1.39e-11 *** |
| rain | 0.031034 | 0.00637 | 4.872 | 1.11e-06 *** |
| aws | 0.271765 | 0.043323 | 6.273 | 3.54e-10 *** |
| vol | 0.013164 | 0.004195 | 3.138 | 0.00170 ** |
| gpCanids | -1.751443 | 0.27253 | -6.427 | 1.30e-10 *** |
| gpFelids | -4.663635 | 0.60911 | -7.656 | 1.91e-14 *** |
| gpMustelids | -2.328113 | 0.22386 | -10.4 | < 2e-16 *** |
| gpUngulates | -0.752006 | 0.256674 | -2.93 | 0.00339 ** |
| gpLagomorphs | -0.51304 | 0.298167 | -1.721 | 0.08531 . |
| gpGround rodents | -0.898842 | 0.19242 | -4.671 | 2.99e-06 *** |
| gpArboreal rodents | -1.012733 | 0.25908 | -3.909 | 9.27e-05 *** |
| year2019 | 0.011537 | 0.240723 | 0.048 | 0.96177 |

Signif. codes: 0 ‘***’ 0.001 ‘**’ 0.01 ‘*’ 0.05 ‘.’ 0.1 ‘ ’ 1

**Supplementary Table 3a |** Modelling stream eDNA species detection in relation to environmental covariates, sampling covariates, and species ecological traits (diet and average weight). Results from the generalized linear model selection procedure are shown in the table below, which presents the list of all models compared. For each model, the table shows: *K,* the number of parameters; *AICc,* the value of the Akaike Information Criteria for small samples; *ΔAICc*, the difference AICc value between the best model and the model evaluated; *AICcWt*, the model weight; and *Cum.Wt*, cumulative *AICc* weight. Model description indicates which covariates were included in the model: *det.ws* is the number of detections per species per day averaged across all camera trap stations present in the watershed, *aws* is the log of the area (km^2^) of the watershed above the sampling site, *vol* is the volume (litre) of water filtered for the sample, *rain* is the precipitation (millimetre) the day prior to the sampling event, and *diet* describes the type of diet of the species and has three levels: animal based (carnivorous strict), mixed animal & plant based (omnivorous), and plant-based (herbivorous).

| Model description | K | AICc | ΔAICc | AICcWt | Cum.Wt |
| --- | --- | --- | --- | --- | --- |
| det.dna~det.ws+rain+aws+vol+diet+wgt | 8 | 1933.17 | 0 | 0.72 | 0.72 |
| det.dna~det.ws+rain+aws+vol+diet+wgt+year | 9 | 1935.06 | 1.88 | 0.28 | 1 |
| det.dna~det.ws+rain+aws+diet+wgt+year | 8 | 1944.23 | 11.05 | 0 | 1 |
| det.dna~det.ws+rain+aws+vol+diet | 7 | 1946.22 | 13.05 | 0 | 1 |
| det.dna~det.ws+rain+aws+vol+diet+year | 8 | 1948.1 | 14.93 | 0 | 1 |
| det.dna~det.ws+aws+vol+diet+wgt | 7 | 1949.85 | 16.67 | 0 | 1 |
| det.dna~det.ws+rain+aws+diet+wgt | 7 | 1950.48 | 17.31 | 0 | 1 |
| det.dna~det.ws+aws+vol+diet+wgt+year | 8 | 1951.75 | 18.57 | 0 | 1 |
| det.dna~det.ws+rain+aws+diet+year | 7 | 1957.19 | 24.01 | 0 | 1 |
| det.dna~det.ws+aws+vol+diet | 6 | 1962.62 | 29.45 | 0 | 1 |
| det.dna~det.ws+rain+aws+diet | 6 | 1963.31 | 30.14 | 0 | 1 |
| det.dna~det.ws+aws+vol+diet+year | 7 | 1964.52 | 31.34 | 0 | 1 |
| det.dna~det.ws+aws+diet+wgt+year | 7 | 1965.56 | 32.38 | 0 | 1 |
| det.dna~det.ws+rain+vol+diet+wgt+year | 8 | 1970.75 | 37.57 | 0 | 1 |
| det.dna~det.ws+rain+diet+wgt+year | 7 | 1976.51 | 43.33 | 0 | 1 |
| det.dna~det.ws+aws+diet+wgt | 6 | 1976.55 | 43.38 | 0 | 1 |
| det.dna~det.ws+aws+diet+year | 6 | 1978.2 | 45.02 | 0 | 1 |
| det.dna~det.ws+rain+vol+diet+year | 7 | 1983.51 | 50.34 | 0 | 1 |
| det.dna~det.ws+rain+vol+diet+wgt | 7 | 1985.34 | 52.17 | 0 | 1 |
| det.dna~det.ws+aws+diet | 5 | 1988.98 | 55.8 | 0 | 1 |
| det.dna~det.ws+rain+diet+year | 6 | 1989.22 | 56.04 | 0 | 1 |
| det.dna~det.ws+vol+diet+wgt+year | 7 | 1990.52 | 57.35 | 0 | 1 |
| det.dna~det.ws+rain+vol+diet | 6 | 1997.95 | 64.78 | 0 | 1 |
| det.dna~det.ws+diet+wgt+year | 6 | 2000.77 | 67.59 | 0 | 1 |
| det.dna~det.ws+vol+diet+year | 6 | 2003.02 | 69.84 | 0 | 1 |
| det.dna~det.ws+vol+diet+wgt | 6 | 2006.92 | 73.74 | 0 | 1 |
| det.dna~rain+aws+vol+diet+wgt | 7 | 2006.98 | 73.81 | 0 | 1 |
| det.dna~rain+aws+vol+diet+wgt+year | 8 | 2008.76 | 75.59 | 0 | 1 |
| det.dna~det.ws+diet+year | 5 | 2013.17 | 80 | 0 | 1 |
| det.dna~rain+aws+vol+diet | 6 | 2016.24 | 83.07 | 0 | 1 |
| det.dna~rain+aws+diet+wgt+year | 7 | 2017.72 | 84.55 | 0 | 1 |
| det.dna~rain+aws+vol+diet+year | 7 | 2018.02 | 84.84 | 0 | 1 |
| det.dna~aws+vol+diet+wgt | 6 | 2018.73 | 85.55 | 0 | 1 |
| det.dna~det.ws+vol+diet | 5 | 2019.23 | 86.05 | 0 | 1 |
| det.dna~aws+vol+diet+wgt+year | 7 | 2020.54 | 87.36 | 0 | 1 |
| det.dna~rain+aws+diet+wgt | 6 | 2022.73 | 89.56 | 0 | 1 |
| det.dna~rain+aws+diet+year | 6 | 2026.91 | 93.73 | 0 | 1 |
| det.dna~aws+vol+diet | 5 | 2027.89 | 94.72 | 0 | 1 |
| det.dna~aws+vol+diet+year | 6 | 2029.7 | 96.53 | 0 | 1 |
| det.dna~rain+aws+diet | 5 | 2031.85 | 98.68 | 0 | 1 |
| det.dna~aws+diet+wgt+year | 6 | 2033.39 | 100.21 | 0 | 1 |
| det.dna~aws+diet+wgt | 5 | 2042.3 | 109.13 | 0 | 1 |
| det.dna~aws+diet+year | 5 | 2042.45 | 109.27 | 0 | 1 |
| det.dna~rain+vol+diet+wgt+year | 7 | 2046.87 | 113.7 | 0 | 1 |
| det.dna~aws+diet | 4 | 2051.27 | 118.1 | 0 | 1 |
| det.dna~rain+diet+wgt+year | 6 | 2052.34 | 119.17 | 0 | 1 |
| det.dna~rain+vol+diet+year | 6 | 2055.85 | 122.67 | 0 | 1 |
| det.dna~rain+diet+year | 5 | 2061.27 | 128.1 | 0 | 1 |
| det.dna~vol+diet+wgt+year | 6 | 2061.35 | 128.18 | 0 | 1 |
| det.dna~rain+vol+diet+wgt | 6 | 2061.94 | 128.77 | 0 | 1 |
| det.dna~det.ws+rain+aws+vol+wgt | 6 | 2064.62 | 131.45 | 0 | 1 |
| det.dna~det.ws+rain+aws+vol+wgt+year | 7 | 2066.5 | 133.33 | 0 | 1 |
| det.dna~vol+diet+year | 5 | 2070.22 | 137.05 | 0 | 1 |
| det.dna~diet+wgt+year | 5 | 2070.58 | 137.41 | 0 | 1 |
| det.dna~rain+vol+diet | 5 | 2070.79 | 137.62 | 0 | 1 |
| det.dna~det.ws+rain+aws+wgt+year | 6 | 2074.61 | 141.44 | 0 | 1 |
| det.dna~vol+diet+wgt | 5 | 2078.04 | 144.87 | 0 | 1 |
| det.dna~diet+year | 4 | 2079.38 | 146.2 | 0 | 1 |
| det.dna~det.ws+rain+aws+wgt | 5 | 2080.13 | 146.96 | 0 | 1 |
| det.dna~det.ws+aws+vol+wgt | 5 | 2080.61 | 147.43 | 0 | 1 |
| det.dna~det.ws+aws+vol+wgt+year | 6 | 2082.5 | 149.33 | 0 | 1 |
| det.dna~vol+diet | 4 | 2086.78 | 153.61 | 0 | 1 |
| det.dna~det.ws+rain+diet+wgt | 6 | 2091.4 | 158.23 | 0 | 1 |
| det.dna~det.ws+aws+wgt+year | 5 | 2095.13 | 161.96 | 0 | 1 |
| det.dna~det.ws+rain+aws+vol | 5 | 2097.41 | 164.24 | 0 | 1 |
| det.dna~det.ws+rain+aws+vol+year | 6 | 2099.3 | 166.12 | 0 | 1 |
| det.dna~det.ws+rain+vol+wgt+year | 6 | 2100.33 | 167.15 | 0 | 1 |
| det.dna~det.ws+rain+diet | 5 | 2103.19 | 170.02 | 0 | 1 |
| det.dna~det.ws+aws+wgt | 4 | 2105.06 | 171.89 | 0 | 1 |
| det.dna~det.ws+rain+wgt+year | 5 | 2105.28 | 172.1 | 0 | 1 |
| det.dna~det.ws+rain+aws+year | 5 | 2107.22 | 174.05 | 0 | 1 |
| det.dna~det.ws+rain+aws | 4 | 2112.56 | 179.39 | 0 | 1 |
| det.dna~det.ws+aws+vol | 4 | 2113.03 | 179.85 | 0 | 1 |
| det.dna~det.ws+rain+vol+wgt | 5 | 2113.94 | 180.76 | 0 | 1 |
| det.dna~det.ws+aws+vol+year | 5 | 2114.91 | 181.74 | 0 | 1 |
| det.dna~det.ws+vol+wgt+year | 5 | 2119.24 | 186.07 | 0 | 1 |
| det.dna~det.ws+aws+year | 4 | 2127.3 | 194.13 | 0 | 1 |
| det.dna~det.ws+wgt+year | 4 | 2128.59 | 195.42 | 0 | 1 |
| det.dna~det.ws+rain+vol+year | 5 | 2132.56 | 199.39 | 0 | 1 |
| det.dna~det.ws+vol+wgt | 4 | 2134.43 | 201.26 | 0 | 1 |
| det.dna~det.ws+aws | 3 | 2136.92 | 203.75 | 0 | 1 |
| det.dna~det.ws+rain+year | 4 | 2137.39 | 204.22 | 0 | 1 |
| det.dna~det.ws+rain+vol | 4 | 2145.91 | 212.73 | 0 | 1 |
| det.dna~det.ws+vol+year | 4 | 2151.1 | 217.93 | 0 | 1 |
| det.dna~det.ws+diet+wgt | 5 | 2159.36 | 226.19 | 0 | 1 |
| det.dna~det.ws+year | 3 | 2160.27 | 227.1 | 0 | 1 |
| det.dna~det.ws+vol | 3 | 2165.98 | 232.8 | 0 | 1 |
| det.dna~rain+diet+wgt | 5 | 2167.49 | 234.32 | 0 | 1 |
| det.dna~det.ws+diet | 4 | 2170.44 | 237.26 | 0 | 1 |
| det.dna~rain+diet | 4 | 2175.63 | 242.46 | 0 | 1 |
| det.dna~rain+aws+vol+wgt | 5 | 2185.48 | 252.3 | 0 | 1 |
| det.dna~rain+aws+vol+wgt+year | 6 | 2187.27 | 254.09 | 0 | 1 |
| det.dna~rain+aws+wgt+year | 5 | 2195.02 | 261.84 | 0 | 1 |
| det.dna~aws+vol+wgt | 4 | 2195.96 | 262.79 | 0 | 1 |
| det.dna~aws+vol+wgt+year | 5 | 2197.76 | 264.59 | 0 | 1 |
| det.dna~rain+aws+wgt | 4 | 2199.23 | 266.05 | 0 | 1 |
| det.dna~aws+wgt+year | 4 | 2209.18 | 276.01 | 0 | 1 |
| det.dna~det.ws+rain+wgt | 4 | 2211.95 | 278.77 | 0 | 1 |
| det.dna~rain+aws+vol | 4 | 2214.75 | 281.58 | 0 | 1 |
| det.dna~rain+aws+vol+year | 5 | 2216.54 | 283.37 | 0 | 1 |
| det.dna~aws+wgt | 3 | 2216.81 | 283.64 | 0 | 1 |
| det.dna~rain+vol+wgt+year | 5 | 2223.61 | 290.43 | 0 | 1 |
| det.dna~rain+aws+year | 4 | 2224.12 | 290.94 | 0 | 1 |
| det.dna~aws+vol | 3 | 2225.02 | 291.85 | 0 | 1 |
| det.dna~diet+wgt | 4 | 2225.7 | 292.53 | 0 | 1 |
| det.dna~aws+vol+year | 4 | 2226.82 | 293.65 | 0 | 1 |
| det.dna~rain+wgt+year | 4 | 2228.15 | 294.98 | 0 | 1 |
| det.dna~rain+aws | 3 | 2228.2 | 295.02 | 0 | 1 |
| det.dna~diet | 3 | 2233.48 | 300.3 | 0 | 1 |
| det.dna~vol+wgt+year | 4 | 2236.62 | 303.45 | 0 | 1 |
| det.dna~rain+vol+wgt | 4 | 2237.98 | 304.8 | 0 | 1 |
| det.dna~aws+year | 3 | 2238.01 | 304.83 | 0 | 1 |
| det.dna~det.ws+rain | 3 | 2242.31 | 309.14 | 0 | 1 |
| det.dna~wgt+year | 3 | 2244.74 | 311.56 | 0 | 1 |
| det.dna~aws | 2 | 2245.45 | 312.27 | 0 | 1 |
| det.dna~rain+vol+year | 4 | 2252.31 | 319.14 | 0 | 1 |
| det.dna~vol+wgt | 3 | 2252.37 | 319.2 | 0 | 1 |
| det.dna~rain+year | 3 | 2256.73 | 323.56 | 0 | 1 |
| det.dna~vol+year | 3 | 2265.09 | 331.91 | 0 | 1 |
| det.dna~rain+vol | 3 | 2266.42 | 333.25 | 0 | 1 |
| det.dna~year | 2 | 2273.03 | 339.85 | 0 | 1 |
| det.dna~det.ws+wgt | 3 | 2276.8 | 343.62 | 0 | 1 |
| det.dna~vol | 2 | 2280.56 | 347.38 | 0 | 1 |
| det.dna~det.ws | 2 | 2306.06 | 372.89 | 0 | 1 |
| det.dna~rain+wgt | 3 | 2335.52 | 402.35 | 0 | 1 |
| det.dna~rain | 2 | 2362.4 | 429.23 | 0 | 1 |
| det.dna~wgt | 2 | 2389.33 | 456.16 | 0 | 1 |
| det.dna~1 | 1 | 2415.38 | 482.21 | 0 | 1 |

**Supplementary Table 3b |** The table shows the beta parameter estimates of the best model: det.dna ~ det.ws + rain + aws + vol + diet + wgt, see Table 3a.

|  | Estimate | Std. Error | z value | Pr(>\|z\|) |
| --- | --- | --- | --- | --- |
| (Intercept) | -6.14759 | 0.347599 | -17.686 | < 2e-16 *** |
| det.ws | 2.828583 | 0.358721 | 7.885 | 3.14e-15 *** |
| rain | 0.027833 | 0.006366 | 4.372 | 1.23e-05 *** |
| aws | 0.257209 | 0.035706 | 7.203 | 5.87e-13 *** |
| vol | 0.012765 | 0.002944 | 4.337 | 1.45e-05 *** |
| dietmixed | 1.614757 | 0.191873 | 8.416 | < 2e-16 *** |
| dietplant | 1.827441 | 0.180765 | 10.109 | < 2e-16 *** |
| wgt | 0.002024 | 0.000515 | 3.928 | 8.58e-05 *** |

Signif. codes: 0 ‘***’ 0.001 ‘**’ 0.01 ‘*’ 0.05 ‘.’ 0.1 ‘ ’ 1

**Supplementary Table 3c |** The table shows the beta parameter estimates of the best model that includes the year effect: det.dna ~ det.ws + rain + aws + vol + diet + wgt + year, see Table 3a.

|  | Estimate | Std. Error | z value | Pr(>\|z\|) |
| --- | --- | --- | --- | --- |
| (Intercept) | -6.2399611 | 0.4314142 | -14.464 | < 2e-16 *** |
| det.ws | 2.8273519 | 0.3587631 | 7.881 | 3.25e-15 *** |
| rain | 0.0278239 | 0.0063611 | 4.374 | 1.22e-05 *** |
| aws | 0.2662895 | 0.0435896 | 6.109 | 1.00e-09 *** |
| vol | 0.0138649 | 0.004237 | 3.272 | 0.00107 ** |
| dietmixed | 1.615022 | 0.1918839 | 8.417 | < 2e-16 *** |
| dietplant | 1.8276244 | 0.1807835 | 10.109 | < 2e-16 *** |
| wgt | 0.0020237 | 0.0005153 | 3.927 | 8.59e-05 *** |
| year2019 | -0.0881001 | 0.2429478 | -0.363 | 0.71688 |

Signif. codes: 0 ‘***’ 0.001 ‘**’ 0.01 ‘*’ 0.05 ‘.’ 0.1 ‘ ’ 1

**Supplementary Table 4 |** Costs of terrestrial mammals’ inventory for each survey method and year (US $). The table below shows all expenses used to estimate the cost of each method. Expenses are classified in four budget categories: *re-usable equipment*, *single use equipment*, *labour and lab work*, and *logistical*. *Re-usable equipment* includes camera traps, water pumps and rechargeable batteries, *single use equipment* includes non-rechargeable AA batteries and eDNA sampling kits, *labour and lab work* combines staff time for field work as well as time spent for data analysis, *logistical* includes expenses related to local transportation from headquarters to field study sites. Transportation costs were calculated by multiplying the per-kilometre cost of using a vehicle by the total number of kilometres required to complete the survey. We used the distance from headquarters to the sampling sites or clusters of sampling sites and the number of trips to obtain a rough estimate of the total distance travelled. We considered an equal total distance for camera trapping in both years and for the eDNA *spatial* design. However, we considered a much lower distance for the eDNA *catchment* design as the two sampling sites were on average closer to the headquarter and length of the survey was reduced (8 days instead of 11).

| **Detail costs for eDNA** | |  |  | 2018 | | 2019 | |
| --- | --- | --- | --- | --- | --- | --- | --- |
| Description | Category | Unit | Unit price | Qty | Total cost | Qty | Total cost |
| Vampire sampler | Reusable equipment | unit | $922 | 1 | $922 |  | $0 |
| Batteries | Reusable equipment | unit | $16 | 8 | $131 |  | $0 |
| Peristatic pump | Reusable equipment | unit | $1,258 |  | $0 | 2 | $2,517 |
| Batteries | Reusable equipment | unit | $75 |  | $0 | 2 | $151 |
| Sampling kit 2018 | Single use equipment | set | $95 | 50 | $4,746 |  | $0 |
| Sampling kit 2019 | Single use equipment | set | $97 |  | $0 | 36 | $3,498 |
| Shipment 2018 | Logistic | lumpsum | $448 | 1 | $448 |  | $0 |
| Shipment 2019 | Logistic | lumpsum | $366 |  | $0 | 1 | $366 |
| Lab work per sample | Labour and lab work | unit | $296 | 50 | $14,803 | 36 | $10,658 |
| Transportation | Logistic | km | $0.50 | 1000 | $500 | 250 | $75 |
| Field work | Labour and lab work | days | $400 | 11 | $4,400 | 8 | $3,200 |
| Total Reusable equipment |  |  |  |  | $1,053 |  | $2,668 |
| Total Single use equipment |  |  |  |  | $4,746 |  | $3,498 |
| Total Logistic |  |  |  |  | $948 |  | $441 |
| Total Labour and lab work |  |  |  |  | $19,203 |  | $13,858 |
| Grand Total |  |  |  |  | $25,950 |  | $20,465 |
|  |  |  |  |  |  |  |  |
|  | | | | | | | |
| **Detail costs for camera trapping** | |  |  | 2018 | | 2019 | |
| Description | Category | Unit | Unit price | Qty | Total cost | Qty | Total cost |
| Camera trap + accessories | Reusable equipment | unit | 210.00 | 57 | $11,970 | 4 | $840 |
| Non-rechargeable AA batteries | Single use equipment | unit | 1.50 | 335 | $503 | 321 | $482 |
| Field work for camera set up | Labour and lab work | days | 400 | 18 | $7,200 | 17 | $6,800 |
| Total transportation for set up | Logistic | km | 0.50 | 1000 | $500 | 1000 | $500 |
| Field work for camera check up | Labour and lab work | days | 400 | 18 | $7,200 | 17 | $6,800 |
| Total transportation for check up | Logistic | km | 0.50 | 1000 | $500 | 1000 | $500 |
| Lab work for image classification | Labour and lab work | days | 400 | 26 | $10,400 | 25 | $10,000 |
| Total Reusable equipment |  |  |  |  | $11,970 |  | $840 |
| Total Single use equipment |  |  |  |  | $503 |  | $482 |
| Total Logistic |  |  |  |  | $1000 |  | $1000 |
| Total Labour and lab work |  |  |  |  | $24,800 |  | $23,600 |
| Grand Total |  |  |  |  | $38,273 |  | $25,922 |

**Supplementary Table 5a |** Modelling site diversity (number of species per sample) in relation to environmental factors. Results from the generalized linear model selection procedure are shown in the table below, which presents the list of all models compared. For each model, the table shows: *K,* the number of parameters; *AICc,* the value of the Akaike Information Criteria for small samples; *ΔAICc*, the difference *AICc* value between the best model and the model evaluated; *AICcWt*, the model weight; and *Cum.Wt*, cumulative *AICc* weight. Model description indicates which covariates were included in the model: *aws* is the area in km2 of the catchment above the sampling site, *vol* is the volume in litre of water filtered for the sample, and *rain* is the precipitation in millimetre the day prior to the sampling event.

| Model description | # par. | | *AICc* | *ΔAICc* | *AICcWt* | *Cum.Wt* | *LL* |
| --- | --- | --- | --- | --- | --- | --- | --- |
| n.sp.dna~aws+rain+vol | | 4 | 416.54 | 0 | 0.73 | 0.73 | -204.02 |
| n.sp.dna~aws+rain+vol+year | | 5 | 418.70 | 2.16 | 0.25 | 0.98 | -203.98 |
| n.sp.dna~aws+rain+year | | 4 | 424.10 | 7.56 | 0.02 | 1 | -207.80 |
| n.sp.dna~aws+rain | | 3 | 427.64 | 11.10 | 0 | 1 | -210.67 |
| n.sp.dna~aws+vol | | 3 | 428.87 | 12.32 | 0 | 1 | -211.29 |
| n.sp.dna~aws+vol+year | | 4 | 430.92 | 14.38 | 0 | 1 | -211.21 |
| n.sp.dna~aws+year | | 3 | 440.59 | 24.05 | 0 | 1 | -217.15 |
| n.sp.dna~aws | | 2 | 447.23 | 30.69 | 0 | 1 | -221.54 |
| n.sp.dna~rain+vol+year | | 4 | 480.95 | 64.41 | 0 | 1 | -236.23 |
| n.sp.dna~rain+year | | 3 | 482.79 | 66.25 | 0 | 1 | -238.25 |
| n.sp.dna~vol+year | | 3 | 496.67 | 80.13 | 0 | 1 | -245.19 |
| n.sp.dna~year | | 2 | 502.59 | 86.05 | 0 | 1 | -249.22 |
| n.sp.dna~rain+vol | | 3 | 510.47 | 93.93 | 0 | 1 | -252.09 |
| n.sp.dna~vol | | 2 | 527.47 | 110.93 | 0 | 1 | -261.66 |
| n.sp.dna~rain | | 2 | 632.53 | 215.99 | 0 | 1 | -314.19 |
| n.sp.dna~1 | | 1 | 701.27 | 284.73 | 0 | 1 | -349.61 |

**Supplementary Table 5b |** The table shows the beta parameter estimates of the best model: n.sp.dna ~ aws + rain + vol, see Table 5a.

|  | Estimate | Std. Error | z value | Pr(>\|z\|) |
| --- | --- | --- | --- | --- |
| (Intercept) | -0.430572 | 0.216331 | -1.99 | 0.046554 * |
| aws | 0.234457 | 0.024634 | 9.517 | < 2e-16 *** |
| rain | 0.014667 | 0.003769 | 3.891 | 9.98e-05 *** |
| vol | 0.006305 | 0.001762 | 3.578 | 0.000346 *** |

Signif. codes: 0 ‘***’ 0.001 ‘**’ 0.01 ‘*’ 0.05 ‘.’ 0.1 ‘ ’ 1

**Supplementary Table 5c |** The table shows the beta parameter estimates of the best model that includes a year effect: n.sp.dna ~ aws + rain + vol + year, see Table 5a.

|  | Estimate | Std. Error | z value | Pr(>\|z\|) |
| --- | --- | --- | --- | --- |
| (Intercept) | -0.481915 | 0.272138 | -1.771 | 0.076586 . |
| aws | 0.239637 | 0.029695 | 8.07 | 7.04e-16 *** |
| rain | 0.014621 | 0.003766 | 3.882 | 0.000103 *** |
| vol | 0.006879 | 0.002561 | 2.686 | 0.007235 ** |
| year2019 | -0.047138 | 0.151572 | -0.311 | 0.755805 |

Signif. codes: 0 ‘***’ 0.001 ‘**’ 0.01 ‘*’ 0.05 ‘.’ 0.1 ‘ ’ 1

SUPPLEMENTARY METHODS

**Supplementary Method 1 |** Estimating the average detection probabilities by species and sampling method.

In order to compare the relative detection power of eDNA vs camera trapping, and among eDNA sampling strategies, we estimated for each species the detection probability per 60-litre water sample for eDNA, and per 60-day for camera trapping. In this analysis, because we were interested in the global pattern of detection at the level of the study area, we assumed the likelihood of detecting a given species to be equivalent across all sites or samples. Below we described the method we used to model the detection probability for camera trapping and eDNA methods.

Model for camera trapping:

Our camera traps were set up on motion detection mode and recorded every species that triggered the sensor over the entire sampling period, therefore the detection probability per species, site and day can be easily estimated from daily detection/non-detection camera trap data. For each species *i*, the total number of days with positive detection $N_{i}$ follows a Binomial distribution:

$$N_{i}\sim Binomial(K,p_{i})$$

where *K* is the number of days and $p_{i}$ is the daily detection probability for the species *i*. Then, the probability of detection for a period of *D* days is given by:

$$P_{i}=1-{(1-p_{i})}^{D}$$

Model for eDNA:

For the eDNA samples, we do not have the detail of the detection/non-detection per litre of water. We only have the information of detection per sample that filtered between 30 and 80 litres of water. Let us consider $r_{i}$ the detection probability of species *i* per litre. If we assume that the detection probability of a given species is equivalent for every litre filtered, then we can write:

$$R_{i}=1-{(1-r_{i})}^{L}$$

with $R_{i}$ the detection probability of a species *i* in a sample of *L* litres of water filtered. Using this equation and the results of detection/non-detection of the species across all samples, we can deduce $r_{i}$, and then recalculate $R_{i}$ for any quantity of water filtered.

We used a Bayesian statistical analysis approach to estimate the detection probabilities of each species with each method and sampling design. All the analyses were performed using program R v3.6.1 (2019) and JAGS software (Plummer 2003) to use Markov Chain Monte Carlo (MCMC) to approximate posterior distributions for every parameter. We assigned a noninformative uniform prior Unif(0,1) to the regression parameter of the detection probability *p*. We recorded 10,000 posterior samples of the model parameters were after 1,000 burn-in iterations. The convergence of the MCMC was monitored by graphical inspection and the method of Gelman and Rubin (1992). All R code, models and data are provided in GitHub (see Code and Data availability section in the manuscript).

REFERENCES

Cantera, I., Cilleros, K., Valentini, A., Cerdan, A., Dejean, T., Iribar, A., Taberlet, P., Vigouroux, R., Brosse, S., 2019. Optimizing environmental DNA sampling effort for fish inventories in tropical streams and rivers. Sci. Rep. 9, 1–11.

Coutant, O., Hansen, C.R., de Thoisy, B., Decotte, J.-B., Valentini, A., Dejean, T., Vigouroux, R., Murienne, J., Brosse, S., 2020. Amazonian mammal monitoring using aquatic environmental DNA. Authorea Prepr.

Plummer, M., others, 2003. JAGS: A program for analysis of Bayesian graphical models using Gibbs sampling, in: Proceedings of the 3rd International Workshop on Distributed Statistical Computing. Vienna, Austria, pp. 1–10.

Pont, D., Rocle, M., Valentini, A., Civade, R., Jean, P., Maire, A., Roset, N., Schabuss, M., Zornig, H., Dejean, T., 2018. Environmental DNA reveals quantitative patterns of fish biodiversity in large rivers despite its downstream transportation. Sci. Rep. 8, 1–13.

R Core Team, 2020. R: A Language and Environment for Statistical Computing. R Foundation for Statistical Computing, Vienna, Austria.
